# Arbitrary Total Angular Momentum Vectorial Holography Using Bi‐Layer Metasurfaces

**DOI:** 10.1002/adma.202519106

**Published:** 2026-02-08

**Authors:** Joonkyo Jung, Hyeonhee Kim, Jonghwa Shin

**Affiliations:** ^1^ Department of Materials Science and Engineering KAIST Daejeon Republic of Korea

**Keywords:** asymmetric transmission, metasurfaces, orbital angular momentum multiplexing, polarization multiplexing, total angular momentum, vector beam multiplexing, vectorial holography

## Abstract

Advanced holographic techniques are increasingly demanded for high‐capacity and secure information processing. In this context, orbital angular momentum (OAM) stands out as a powerful resource for optical multiplexing, offering access to an unbounded set of orthogonal modes. To harness this potential, metasurfaces, with their considerable ability to control light, have emerged as key platforms for OAM‐multiplexed holography. Nevertheless, conventional OAM holography suffers from limited polarization engineering capabilities due to the lack of chirality control in single‐layer metasurfaces. Here, we introduce a bi‐layer metasurface architecture that realizes total angular momentum (TAM) vectorial holography, where TAM represents the combination of spin angular momentum (SAM, equivalent to polarization) and OAM of light. In contrast to previous approaches, this scheme enables true polarization–OAM multiplexing, facilitating the independent generation of vectorial holographic images for each orthogonal TAM input state. This concept is validated numerically and experimentally, confirming the feasibility of TAM vectorial holography. The proposed scheme can be easily integrated with other recent holography generation approaches, such as vector beam multiplexing and bidirectional holography, thereby further expanding its multiplexing capability. This work establishes a versatile framework for advanced full‐vectorial holography, showing how metasurfaces can unlock multiplexing strategies for emerging photonic systems.

## Graphical abstract

A bi‐layer metasurface platform is presented for arbitrary total angular momentum vectorial holography, where polarization and orbital angular momentum are exploited as independent multiplexing dimensions. By overcoming symmetry limitations of single‐layer designs, the approach generates distinct vectorial holographic images for each orthogonal TAM state. The concept is numerically and experimentally validated and extended to vector‐beam and bidirectional holography.

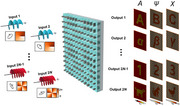

## 1 Introduction

Optical holography offers a powerful means of recording and reconstructing optical fields while preserving their exact properties, including amplitude, phase, and polarization. This enables highly realistic 3D displays as well as high‐density optical data storage [1, 2, 3]. With rising demand, advanced holographic techniques—particularly optical multiplexing, the intentional encoding of independent information channels across multiple physical degrees of freedom of light—have emerged as key research directions for increasing data capacity, reducing device size, and inducing new functionalities [4, 5, 6, 7, 8, 9, 10, 11, 12, 13, 14, 15]. The applications of optical multiplexing extend beyond holography, driving breakthroughs in high‐speed optical communications [16, 17, 18, 19], quantum information processing [20, 21], and optical computing architectures [22, 23], establishing it as a vibrant research frontier across photonics.

Among the potential degrees of freedom that can be harnessed, orbital angular momentum (OAM), characterized by a helical phase front, has attracted considerable interest due to its unique property of supporting an unbounded set of orthogonal helical modes, thereby providing a promising pathway toward highly multiplexed holographic systems [24, 25, 26]. In this scheme, different sets of information are encoded according to the helical mode index, which specifies the number of 2*π* phase twists around the beam axis, allowing the reconstruction of independent optical fields for each OAM state of the incident beam. Despite this intriguing idea, conventional bulk optics and spatial light modulators utilized for OAM holography impose limitations on device performance due to their inherent technological hurdles. Notably, their low spatial resolution restricts the achievable fidelity and channel capacity of OAM‐multiplexed holograms [24]. Moreover, combining OAM with other optical degrees of freedom, such as the polarization states of incident and outgoing light, remains challenging.

To overcome these limitations, metasurfaces—planar arrays of nanostructures with subwavelength dimensions—have recently emerged as promising platforms for next‐generation optical devices [27, 28, 29]. By individually modulating the shape and dimensions of the constituent nanostructures, metasurfaces can tailor their optical responses, such as amplitude gains, phase delays, and polarization conversions, with subwavelength spatial resolution. In addition, they can be designed to behave differently depending on the configuration of the incident light [28, 30, 31, 32, 33, 34], offering various forms of optical multiplexing at minimal additional cost. This versatility renders metasurfaces a highly adaptable platform for further advancing OAM holography.

**FIGURE 1:**
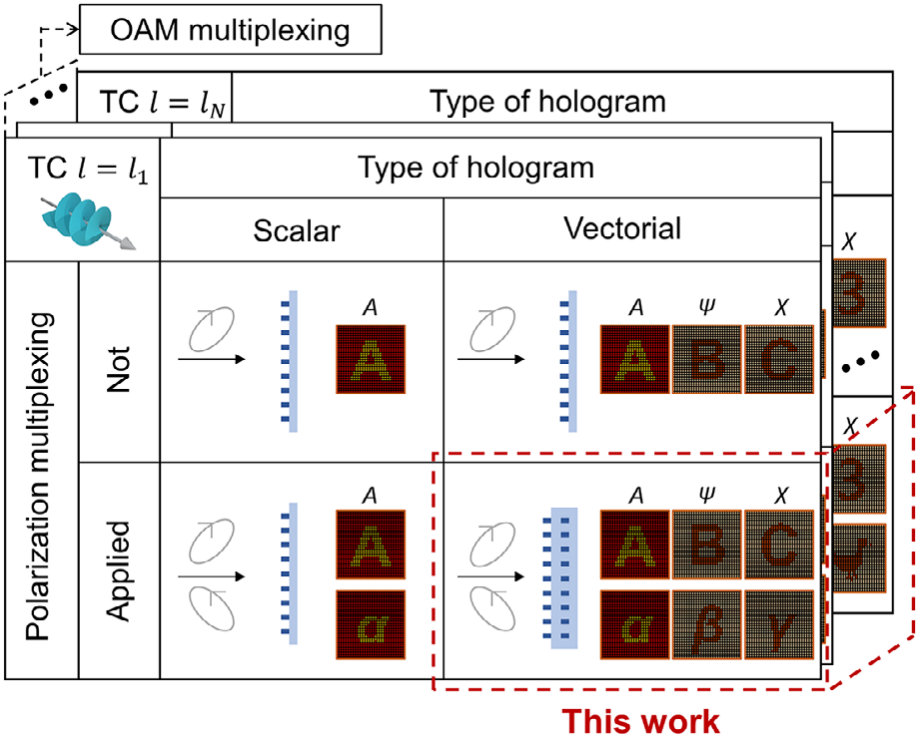
Classification of OAM holography. Comparison of OAM holography in terms of hologram type (scalar or vectorial) and polarization‐multiplexing capability. The proposed approach achieves vectorial holography with polarization–OAM multiplexing.

In particular, the arbitrarily designable anisotropy of metasurfaces provides high degrees of freedom in polarization control and has given rise to two important subfields of OAM holography: polarization‐multiplexed OAM (scalar) holography [35, 36, 37, 38, 39, 40, 41] and OAM vectorial holography with a single predefined incident polarization for each OAM state [41]. In polarization‐multiplexed OAM holography, different holograms can be realized with two independent multiplexing dimensions of the incident polarization and OAM, enabling the production of holographic images with designable intensities. In OAM vectorial holography, distinct vectorial holographic images with engineered intensity and polarization distributions can be reconstructed for given OAM states, as schematically represented in Figure 1. In [41], such vectorial OAM holography was realized using a single‐layer metasurface. This work represents a significant step beyond earlier scalar OAM holography and highlights the potential of carefully engineered single‐layer platforms for vectorial light manipulation.

Nevertheless, current implementations based on single‐layer metasurfaces face intrinsic limitations in polarization manipulation because they lack the controllability of the chirality of the system. Specifically, the single‐layer metasurface architecture inherently preserves mirror symmetry along the depth direction, which imposes symmetry constraints on the Jones matrix and prevents polarization from functioning as an independent multiplexing dimension for vectorial wavefront manipulation [42]. As a result, this structural symmetry fundamentally hinders the realization of fully arbitrary polarization‐multiplexed vectorial holography within a single‐layer architecture.

In the context of OAM holography, this limitation manifests itself as a trade‐off between polarization multiplexing and full‐vector designability. With current single‐layer metasurfaces, polarization‐multiplexed OAM holograms can reproduce the target holographic images only in their intensity patterns and not in their polarization distributions, whereas vectorial OAM holograms can be designed only for a single input polarization for each OAM state. In other words, one must choose either the polarization multiplexing capability or full‐vector designability, but not both. Importantly, this intrinsic limitation cannot be overcome simply by changing the shape or dimensions of the nanostructures. Unifying these two subfields and realizing polarization‐multiplexed, fully vectorial OAM holography mandates the introduction of new principles.

In this study, we propose a new approach to realize arbitrary total angular momentum (TAM) vectorial holography. In this approach, each TAM state—defined by a polarization state (i.e., spin angular momentum, SAM) and an OAM mode of the incident light—is mapped to a distinct output vector field as shown in Figure 1, thereby establishing incident polarization and OAM as independent multiplexing dimensions for vectorial holography. This is enabled by leveraging bi‐layer metasurface architectures, previously reported by our group [42], which overcome the symmetry constraints of conventional single‐layer designs, thereby enabling complete control over anisotropy and chirality independently (refer to Text S1 for the detailed comparison between single‐layer and bi‐layer metasurfaces). Furthermore, we introduce a unified framework to optimize metasurfaces considering all TAM channels in a holistic manner. We numerically and experimentally demonstrate the proposed fully polarization–OAM‐multiplexed TAM vectorial holography principle with examples. In addition, we propose two new holographic configurations enabled by integrating our proposed scheme into other state‐of‐the‐art holographic multiplexing mechanisms: (1) vector beam‐multiplexed vectorial holography and (2) bidirectional TAM vectorial holography. Our results establish a generalizable platform for TAM‐multiplexed vectorial holography, paving the way toward TAM‐enabled, highly multiplexed optical devices.

## 2 Results and Discussion

### 2.1 Arbitrary TAM Vectorial Holography

In contrast to conventional holography, where the illumination beam is typically a simple plane wave, OAM‐“preserving” holography can utilize complex illuminations with non‐trivial OAM; it can then reproduce the incident beam's OAM characteristics at each reconstructed image pixel, which is discretely sampled over the target image in a regular grid pattern. A related, but different configuration that embeds the conjugate phase of an input beam with the target OAM enables OAM‐“selective” holography, where the metasurface cancels the helical phase of the target input beam and generates fundamental Gaussian‐like beams with zero OAM that have local intensity maxima at the center of each image pixel. Since other input beams with different OAM are converted by the metasurface to beams with non‐zero OAMs and a singular point (zero intensity) at the center of each pixel, simple spatial filtering with a pinhole‐like aperture array can reject the other inputs. Furthermore, by designing a metasurface with multiple OAM‐selective holographic phase profiles superimposed, multitudes of images can be embedded, with each image uniquely activated by the corresponding input beam with the correct OAM. This provides a systematic way of OAM‐“multiplexed” holography.

**FIGURE 2:**
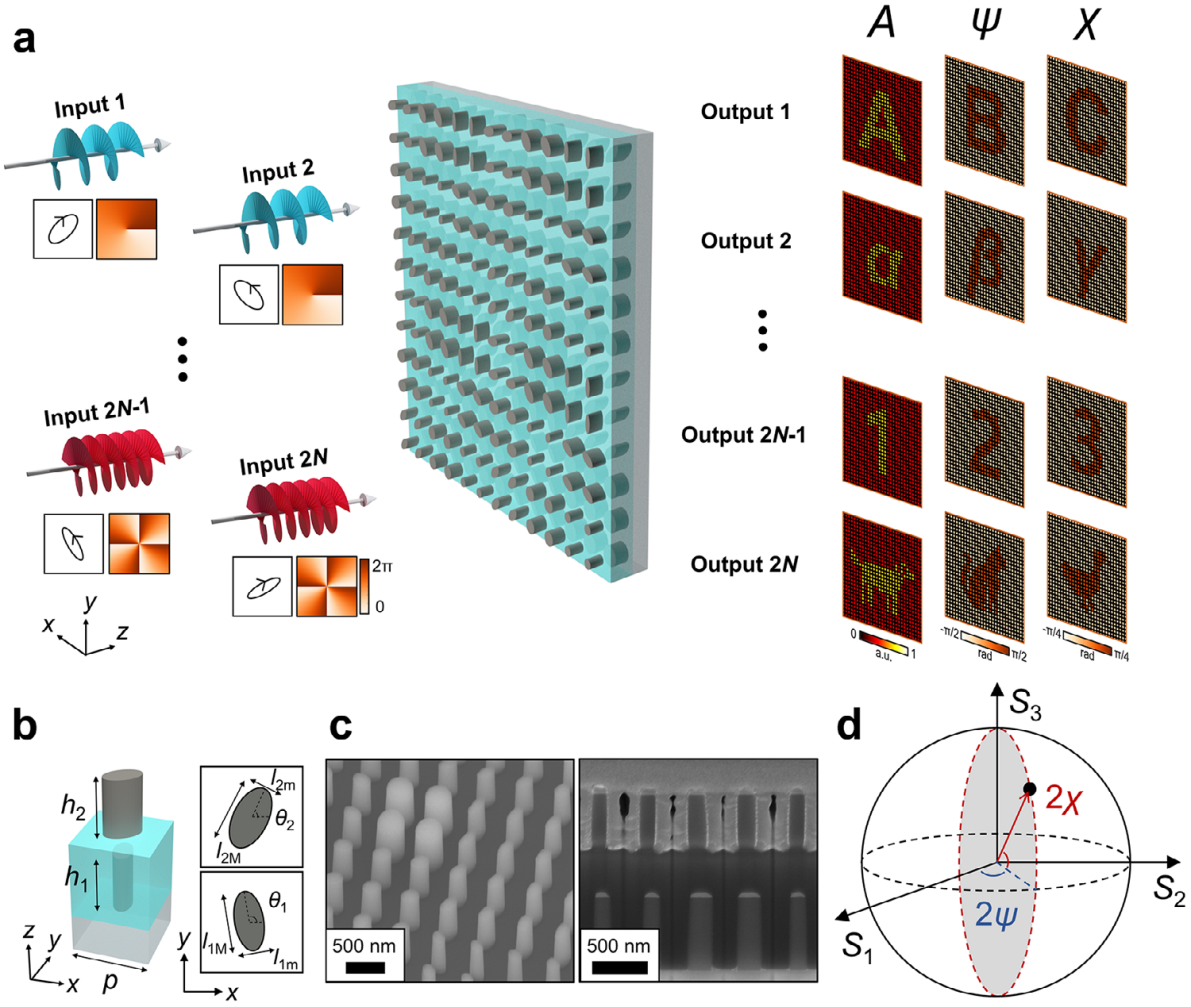
TAM vectorial holography. (a) Schematic of the TAM vectorial holography concept. Polarizations and phases, defining input TAM states, are represented in the square box. Three target images for each output channel are encoded using the intensity (*A*), azimuth (*ψ*), and elevation (*χ*). (b) Unit cell structure of the bi‐layer metasurface. The right panels show cross sections of the top (the subscript 2) and bottom (the subscript 1) layers of the bi‐layer structure. *p*: period; *h*: height of posts; *l*_M,m_: length of the major (M) and minor (m) axes; *θ*: orientation angle. (c) Scanning electron microscopy images of the fabricated bi‐layer metasurface. (d) Representation of azimuth and elevation for image encoding on the Poincaré sphere.

Building on these principles, we extended the concept to TAM vectorial holography by exploiting the polarization multiplexing and manipulation capabilities of bi‐layer metasurfaces. Figure 2 presents an overview of the proposed TAM vectorial holography device, which generates independent sets of 2D point‐like target holographic images according to the TAM input states. In the schematics in Figure 2a, each TAM input state |*p*, *l*〉 can be defined by its polarization *p* and helical mode index *l*. As shown in the right columns of Figure 2a, the regions masked by the aperture array are depicted in black throughout this work. The bi‐layer metasurface, with its unit cell schematics and scanning electron microscopy (SEM) images shown in Figure 2b,c, transforms each TAM state into the desired vectorial holographic images. Three target images per output channel are presented to visualize their independent designability. These images correspond to the far‐field intensity and polarization profiles, with the latter represented by two spherical coordinates (azimuth and elevation angles) of polarization on the Poincaré sphere (Figure 2d). For multiplexing, orthogonal TAM input states were considered; two TAM input states are orthogonal (〈*p*_1_, *l*_1_|*p*_2_, *l*_2_〉 = 0) when their polarizations are orthogonal (〈*p*_1_|*p*_2_〉 = 0) or their helical mode indices differ ($l_{1}\neq l_{2}$). Notably, our scheme accommodates arbitrary elliptical polarization states as input polarizations.

Recent advancements in metasurfaces have widened the controllability of the amplitude, phase, and polarization state of light. In particular, bi‐layer dielectric metasurfaces have been demonstrated to achieve complete linear control over coherent light transmission [42, 43, 44]. Based on the Jones calculus [45], we represented our bi‐layer metasurfaces as unitary Jones matrices, where unitarity simplifies the design process and guarantees the maximal power efficiency of the device. Various factors, such as material absorption and fabrication imperfections, may result in non‐unitarity and a decrease in efficiency. Unitary matrices can be further decomposed using four design parameters *θ*_1_, *θ*_2_, *ϕ*_1M_, and *ϕ*_1m_ given as [42]:

(1) $$\begin{array}{c} U=\left[\begin{array}{cc} \cos\left(2\theta_{2}-\theta_{1}\right) & \sin\left(2\theta_{2}-\theta_{1}\right)\\ \sin\left(2\theta_{2}-\theta_{1}\right) & \cos\left(2\theta_{2}-\theta_{1}\right) \end{array}\right]\left[\begin{array}{cc} e^{i\phi_{1M}} & 0\\ 0 & e^{i\phi_{1m}} \end{array}\right]\left[\begin{array}{cc} \cos\theta_{1} & \sin\theta_{1}\\ -\sin\theta_{1} & \cos\theta_{1} \end{array}\right] \end{array}$$

These design parameters are closely related to the structural parameters of the bi‐layer unit cell in Figure 2b; *θ*_*i*_ corresponds to the orientation angles of the major axes of the elliptical posts of the bottom (*i* = 1) and top (*i* = 2) layers, and *ϕ*_1M,1m_ are the phase delays of the bottom layer along the major and minor axes, which are directly related to the lengths of the major and minor axes *l*_1M,1m_ of the bottom layer. The lengths of the major and minor axes *l*_2M,2m_ of the top layer were fixed across the entire metasurface so that all the nanoposts on the top layer acted as local half‐wave retarders with spatially varying rotation angles (Text S2). Further quantitative analyses of the interlayer alignment tolerance and layer‐resolved dispersive transmission characteristics of bi‐layer structures, which are critical for their practical implementation, are provided in Text S3 and S4.

To realize a unified optimization framework that simultaneously incorporates all TAM channels within a single design flow, we adopt a joint optimization approach that directly optimizes the Jones matrix of the metasurface. Unlike conventional OAM holography, where phase‐only holograms are independently optimized in a channel‐wise manner for predefined input–output polarization channels (Text S5), our approach enables the holistic optimization of vectorial holograms across all TAM channels. By directly optimizing the Jones matrix, the proposed framework allows a broader design space to be explored than phase‐only, channel‐wise hologram optimization.

**FIGURE 3:**
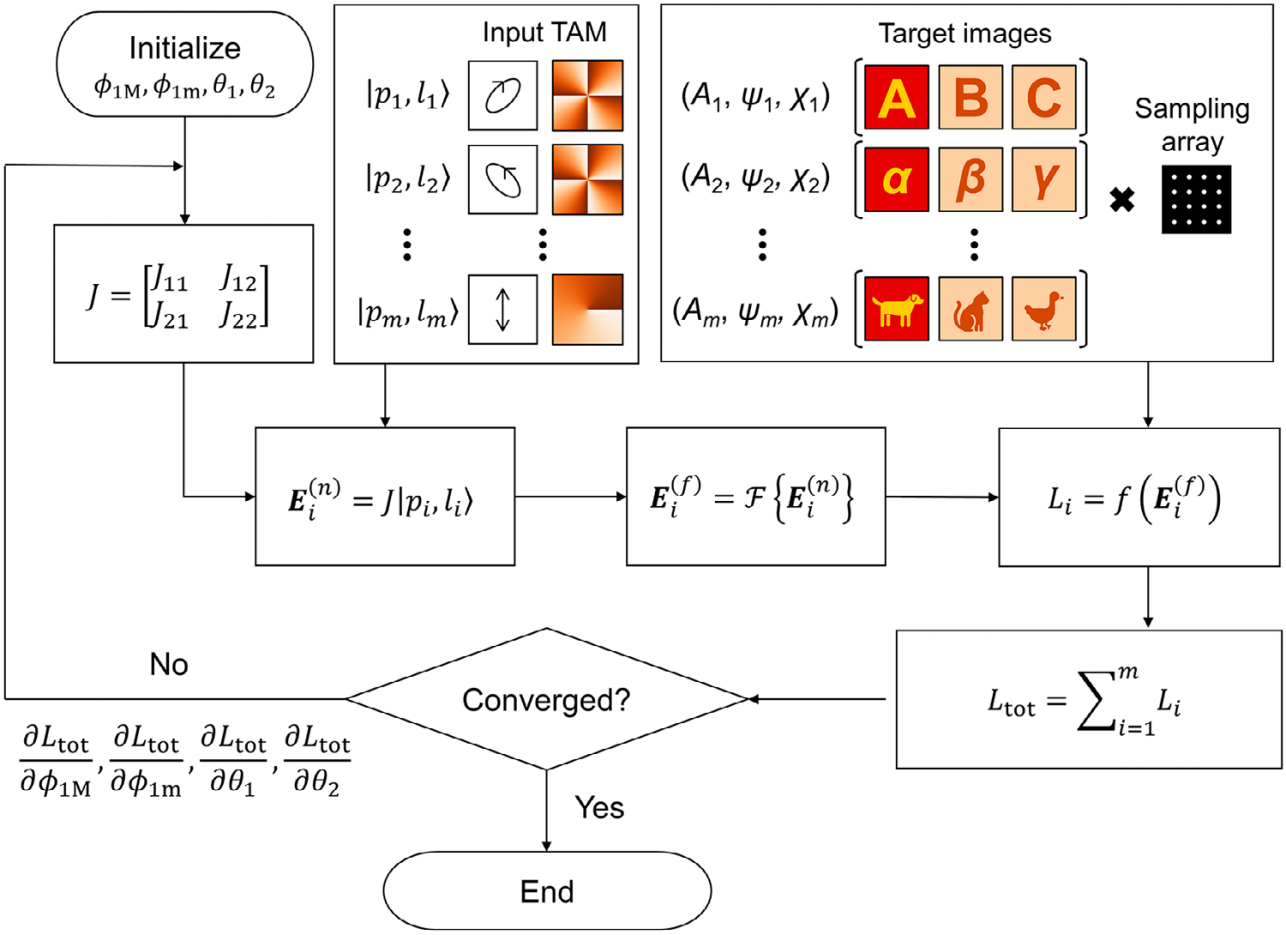
Optimization workflow for TAM vectorial holography. Flowchart of the gradient descent optimization used to optimize bi‐layer metasurface parameters.

This framework is implemented using a gradient descent optimization algorithm [46, 47]. Given TAM input states and sets of target images discretized by a 2D sampling array, random initial parameters are iteratively updated by minimizing the difference between the calculated far‐field profiles and the targets, as illustrated in the optimization flowchart shown in Figure 3. During optimization, partial derivatives are analytically computed using the chain rule (Text S6). Within this framework, multiplexing across multiple TAM channels can be readily achieved by incorporating additional input states and corresponding target vectorial images into the optimization. In practice, the achievable multiplexing performance is influenced by physical and design‐related constraints, and the associated crosstalk behavior is discussed in Text S7.

**FIGURE 4:**
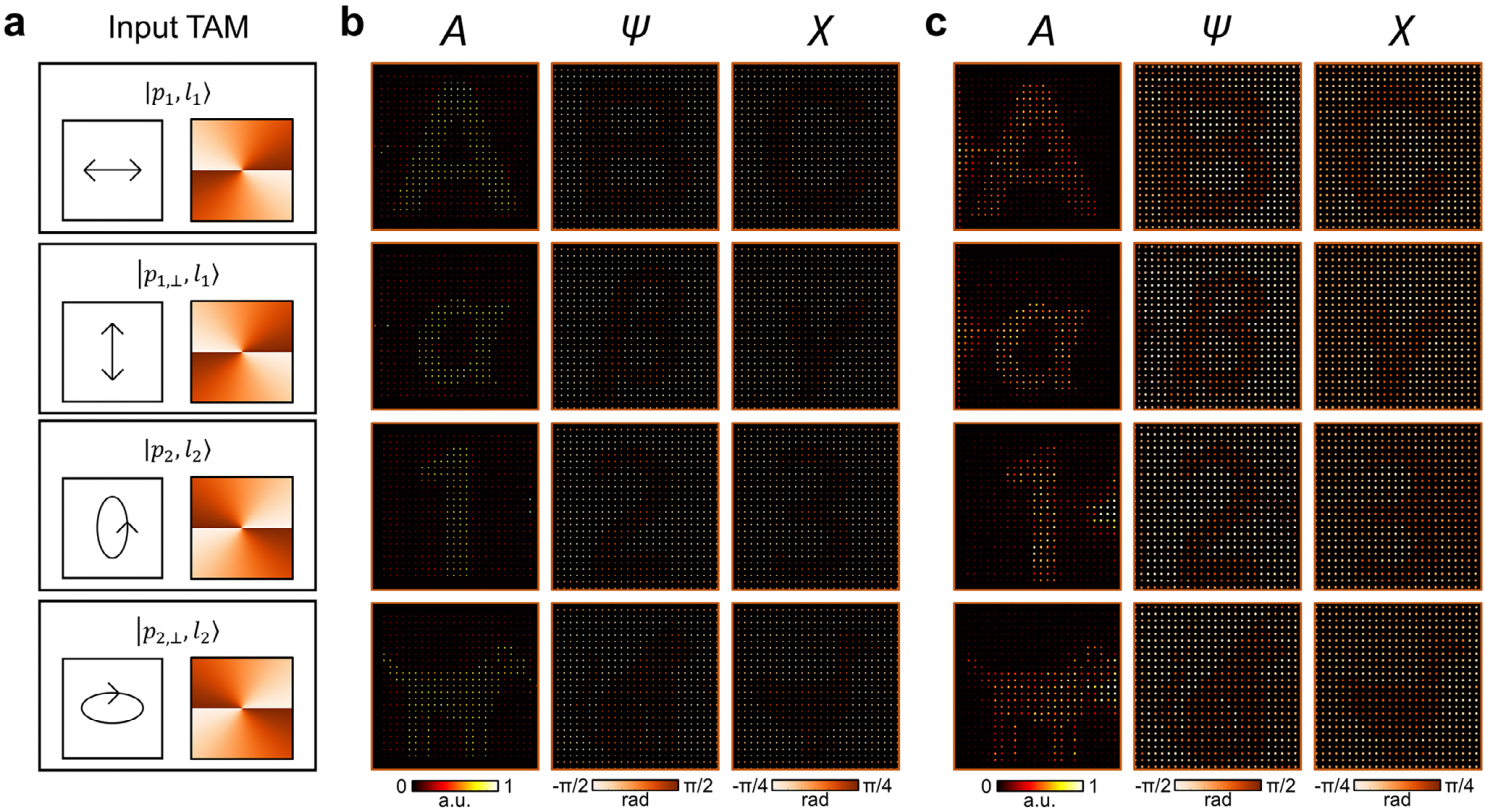
Numerical and experimental demonstrations of TAM vectorial holography. (a) Polarizations and phases of the four input TAM states. (b) Numerically reconstructed holographic images. (c) Experimentally reconstructed holographic images.

Figure 4 summarizes the numerical and experimental demonstrations of the proposed TAM vectorial holography. For the demonstrations, we designed a metasurface that produces 12 scalar (four vectorial) holographic images in response to four different TAM input states. Figure 4a depicts the chosen four TAM states with the polarization states of |*p*_1_〉 = [1; 0], |*p*_1,⊥_〉 = [0; 1], $|p_{2}\rangle =\left[\cos\frac{\pi }{3};\;e^{i\frac{\pi }{2}}\sin\frac{\pi }{3}\right]$, and $|p_{2,⊥}\rangle =\left[-\sin\frac{\pi }{3};\;e^{i\frac{\pi }{2}}\cos\frac{\pi }{3}\right]$ and the helical mode indices of *l*_1_ = −2 (for |*p*_1_〉 and |*p*_1,⊥_〉) and *l*_2_ = 2 (for |*p*_2_〉 and |*p*_2,⊥_〉), respectively. Each TAM input state was intended to generate different sets of three scalar images in its intensity and polarization distributions: Latin letters (“A,” “B,” and “C”), Greek letters (“α,” “β,” and “γ”), numbers (“1,” “2,” and “3”), and animal icons (dog, cat, and duck), respectively (refer to Figure S6 for the ground truth images). In practice, these three scalar images for each TAM input would be designed such that a single fully vectorial target holographic image with coupled intensity and polarization distributions is formed as a result. However, in such cases, a high degree of correlation typically exists between the three images. Thus, to demonstrate their independent designability even more clearly, we specifically chose these examples such that each scalar image was completely different from the others.

For easy visual inspection of the reproduction quality, target images were binarized for intensity (*A* = 0.67, 0.33), azimuth (*ψ* = ±*π*/4), and elevation (*χ* = ±*π*/8). However, the design method itself supports arbitrary, non‐binarized target values. To apply OAM multiplexing, a 2D sampling function was used to discretize the target images in a square lattice with a period of 0.08 in the normalized spatial‐frequency domain. For clear visualization, the output channels for different helical mode indices were spatially separated (refer to Text S8 for the detailed configuration). Although the experimental demonstrations are presented for four TAM input states, this choice does not indicate a performance limit of the proposed approach. To verify its scalability, we additionally designed and numerically evaluated a metasurface capable of generating 48 scalar (16 vectorial) holographic images in response to 16 different TAM input states, as demonstrated in Figure S8.

Figure 4b presents the numerical reconstruction of the holographic images, which clearly reproduce the intended intensity and polarization distributions for each TAM input state. The agreement between the reconstructed and target images was evaluated across all 12 images (intensity and polarization), yielding an average correlation coefficient of ≈0.962. Polarization fidelity was further assessed from the cosine similarity of the Stokes parameter distributions of the four output channels, giving an average value of ≈0.963. These results confirmed the high fidelity of the numerical design (refer to Experimental Section for definitions of these measures). The optimized diffraction efficiencies of the four output channels, defined as the ratio of the power passing through the aperture array within the target region to the incident power, were ≈19.5%, 16.7%, 16.6%, and 18.9%, respectively, assuming unitary transmission. Note that the diffraction efficiencies are strongly influenced by the number of encoded helical modes in the OAM multiplexing mechanism and the diameter of apertures in the aperture array (Text S9).

Figure 4c shows the experimental reconstruction of the holographic images. Details of the post‐processing procedure for the measured images can be found in Text S10. The optimized design was fabricated using well‐established electron beam lithography and reactive‐ion etching, and was optically characterized using a Stokes parameter measurement setup (Experimental Section). For these measurements, an input‐generating metasurface was positioned directly in front of the hologram. A detailed description of these input‐generating metasurfaces and the optical characterization configurations can be found in Text S11. The close match between the simulation and experiment (Figure 4b,c) clearly demonstrates the feasibility of the proposed TAM vectorial holography. This proves that a fully vectorial target holographic image can be realized for each TAM input state, even when some of the input states share the same OAM and differ only in their polarizations, which is impossible with previous single‐layer metasurfaces.

### 2.2 Vector Beam‐Multiplexed Vectorial Holography

When multiple TAM states are collinearly superposed, the resulting field forms a generalized vector beam (VB) with complex polarization and phase distributions. The simplest case is the superposition of two TAM states. Such a vector beam |*ψ*〉 can be represented as |*ψ*〉 = *α*|*p*_1_,*l*_1_〉 + *β*|*p*_2_,*l*_2_〉, where *α* and *β* are the complex amplitudes of the constituent states. The detailed beam profile is determined based on their relative magnitudes and phase differences.

**FIGURE 5:**
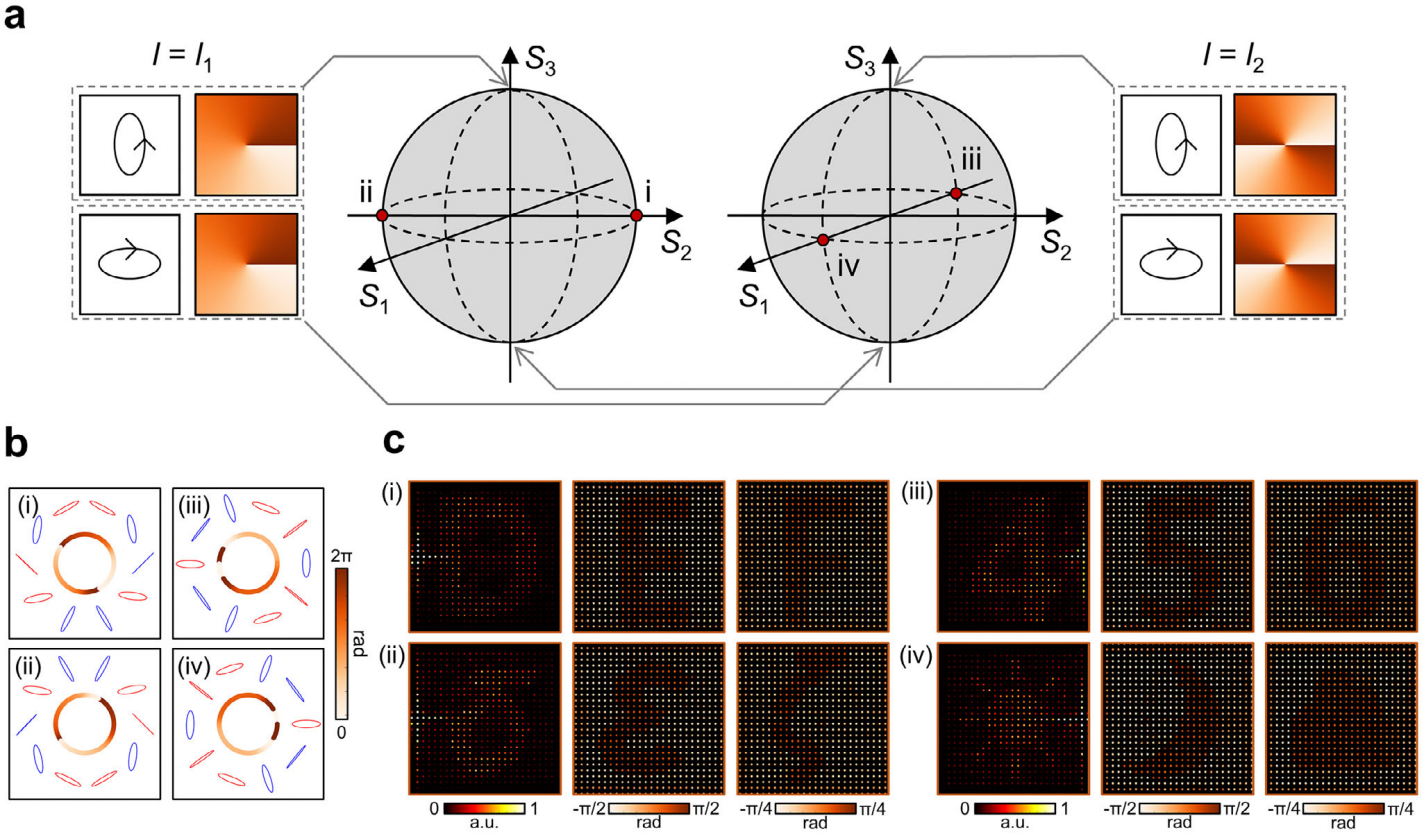
Vector beam‐multiplexed TAM vectorial holography. (a) Higher‐order Poincaré spheres representing the vector beam (VB) space. Each sphere is defined by two TAM states with a pair of orthogonal polarizations *p* and *p*_⊥_ and different helical mode indices *l*_1_ and *l*_2_. Red dots indicate the selected input VBs. (b) Polarization and phase profiles of the selected input VBs. (c) Experimental reconstructions of VB‐multiplexed vectorial holography.

A higher‐order Poincaré sphere (HOPS) offers a concise visualization of all possible VBs [48]. In the HOPS representation (Figure 5a), the two constituent TAM states with potentially different OAMs ($l_{1}\neq l_{2}$) correspond to the north and south poles. This generalizes the conventional Poincaré sphere, in which the north and south poles are usually circular polarization states, and no explicit consideration for OAM is given. Similar to the conventional Poincaré sphere, the elevation and azimuth represent the relative magnitudes and phase differences of the two TAM states corresponding to both poles. Thus, every point on the sphere corresponds to a unique VB state.

The use of generalized VBs instead of simple TAM states extends the input degrees of freedom in optical holography [38, 39, 40]. The proposed VB‐multiplexed vectorial holography generates a target vectorial hologram only when the correct VB is incident on the device. Previous VB‐based multiplexing approaches were limited in polarization diversity, and only one VB state per HOPS with different polarizations and helical mode indices could be employed for multiplexing vectorial holograms due to the lack of controllability of light transmission [41]. By contrast, we rigorously validated the extended capability of our approach by deliberately choosing VBs that were as similar to one another as possible without violating orthogonality, thereby demonstrating the independent designability of the resulting vectorial holograms even in such challenging cases. As a result, our design enables the maximum number of two independent input states to be selected from a single HOPS, so that four VBs can be utilized as input states from just two HOPSs instead of four. Furthermore, we put additional constraints on the two HOPSs so that they are as closely related to each other as possible: their polarization states on the north and south poles are identical between HOPSs and only their helical mode numbers were flipped (i.e., the north and south poles have *l*_1_ and *l*_2_ in the first HOPS, and *l*_2_ and *l*_1_ in the second). Although we used two TAM states to form each VB, this concept can be readily extended to more complex VBs composed of three or more TAM states.

Figure 5 shows the experimental realization of VB‐multiplexed vectorial holography. We designed and fabricated a metasurface sample based on the same procedures described in the previous section, but with VB input states. The constituent TAM states had polarization $\left|p\right\rangle =\left[\cos\frac{\pi }{3};\;e^{i\frac{\pi }{2}}\sin\frac{\pi }{3}\right]$ and helical mode indices *l*_1_ = −1, *l*_2_ = 2. The first HOPS comprised |*p*, *l*_1_〉 and |*p*_⊥_, *l*_2_〉, and the second |*p*, *l*_2_〉 and |*p*_⊥_, *l*_1_〉 (Figure 5a). Without loss of generality, we chose two VB states corresponding to a pair of antipodal points from each HOPS (i.e., four VB states from four TAM states), marked by red dots on each HOPS in Figure 5a. Each input VB was intended to generate different categories of scalar images, in a similar manner with the previous section: Latin letters (“D,” “E,” and “F”), Greek letters (“δ,” “ε,” and “ζ”), numbers (“4,” “5,” and “6”), and weather icons (sun, moon, and cloud), respectively (refer to Figure S6 for the ground truth images). Figure 5b shows the polarization and phase distributions of the chosen VBs. As the constituent TAM states were assumed to have uniform intensity distributions and orthogonal polarizations, the resulting VBs exhibited uniform intensity distributions with radially constant polarization and phase distributions.

To prepare the desired VB inputs, we fabricated input‐generating metasurfaces (Text S11). Figure 5c presents the post‐processed experimental results, which clearly reconstruct the target intensity and polarization images. These results agree well with the numerical predictions (Figure S12), confirming the effectiveness of the proposed VB‐multiplexed vectorial holography. Note that the additional multiplexing dimensions provided by this approach can significantly enhance the versatility of metasurface‐based holography and enable new possibilities in applications such as new and more secure encryption schemes in optical communication [46, 49, 50].

### 2.3 Bidirectional TAM Vectorial Holography

The bidirectional asymmetric transmission of light, where an optical system produces distinct optical responses depending on the illumination direction, holds considerable potential for expanding optical functionalities [45, 51, 52, 53]. Our recent work demonstrated that bi‐layer metasurfaces can realize polarization–direction‐multiplexed vectorial holograms, in which different vectorial holographic images are generated depending on both the illumination direction and polarization state [47]. Despite the potential benefits of bidirectionality, its combination with OAM holography has rarely been explored (with one exception in the field of nonlinear OAM holography [54]). In this section, we propose bidirectional TAM vectorial holography that seamlessly integrates bidirectionality with TAM vectorial holography to add a new dimension to the multiplexing capability of metasurface‐based holography.

**FIGURE 6:**
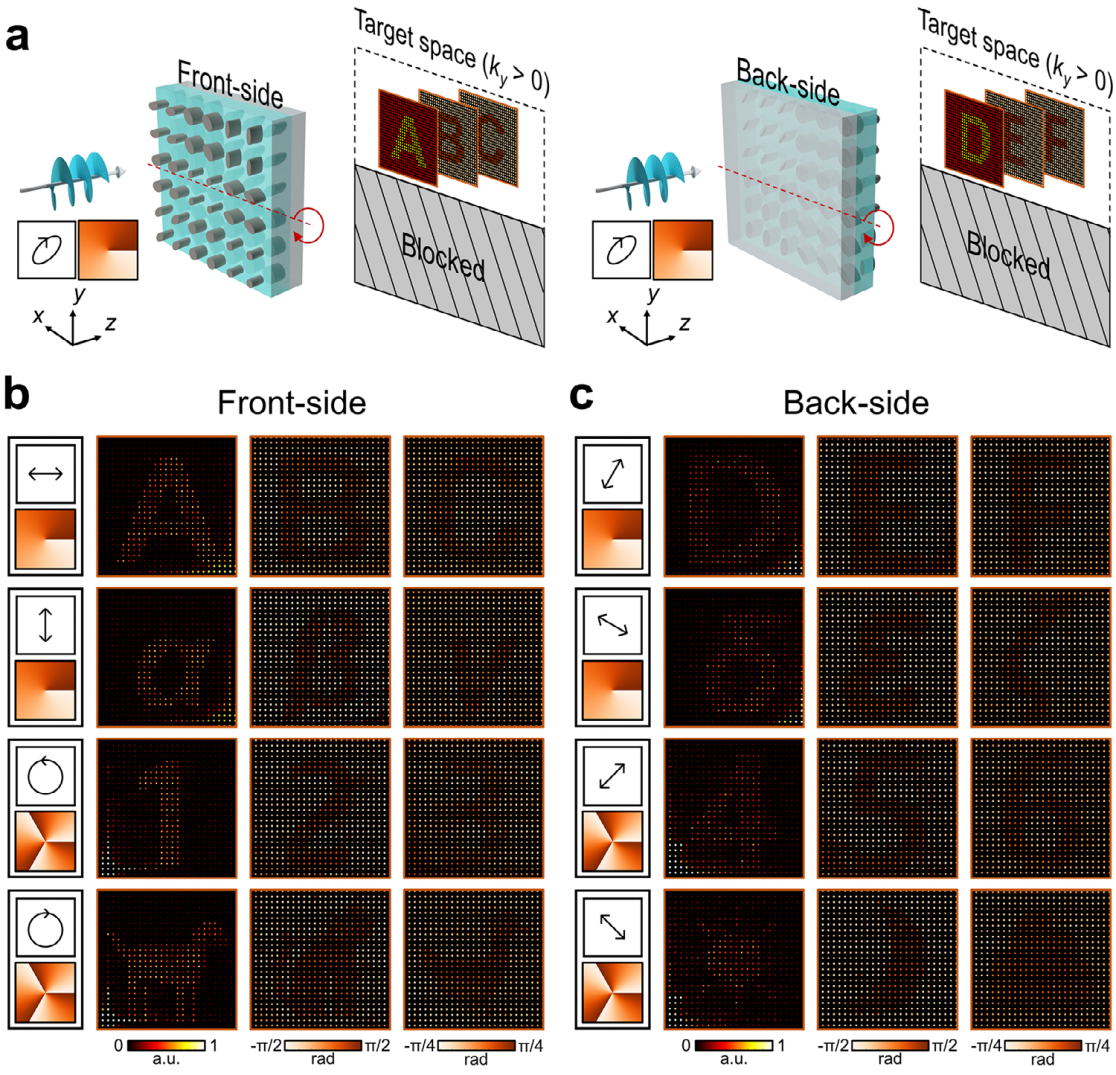
Bidirectional TAM vectorial holography. (a) Conceptual schematic of bidirectional operation, where the transmission space is partitioned, and the lower half‐space is blocked. The metasurface sample is rotated about the *x*‐axis to switch the direction of illumination. Experimental reconstructions for (b) front‐side illumination and (c) back‐side illumination demonstrate the TAM–direction‐multiplexing capability of bidirectional TAM vectorial holography. The leftmost column in panels (b) and (c) shows the incident polarization and OAM states.

Figure 6 presents the conceptual schematics and experimental results of the proposed approach. To obtain independent TAM–direction‐multiplexed vectorial holograms, the transmission space was spatially partitioned, with the lower half‐space excluded; this can be implemented either by physically blocking the lower half‐space or by restricting the measurement to a region of interest corresponding to the upper half‐space (Figure 6a) [47]. Using the proposed design method, with a minor adaptation that simultaneously considers illumination from both directions, we designed and fabricated a metasurface sample to demonstrate bidirectional TAM vectorial holography. For front‐side illumination, the four chosen TAM states were |*p*_1_, *l*_1_〉, |*p*_1,⊥_, *l*_1_〉, |*p*_2_, *l*_2_〉, and |*p*_2,⊥_, *l*_2_〉, where |*p*_1_〉 = [1; 0], $|p_{2}\rangle =\frac{1}{\sqrt{2}}\left[1;\;i\right]$, *l*_1_ = −1, and *l*_2_ = 3. For back‐side illumination, the chosen TAM states were |*p*_3_, *l*_1_〉, |*p*_3,⊥_, *l*_1_〉, |*p*_4_, *l*_2_〉, and |*p*_4,⊥_, *l*_2_〉 where $|p_{3}\rangle =\left[\cos\frac{\pi }{3};\sin\frac{\pi }{3}\right]$, $|p_{4}\rangle =\frac{1}{\sqrt{2}}\left[1;\;1\right]$. Each TAM input state was designed to generate different categories of images: Latin letters (“A,” “B,” and “C”), Greek letters (“α,” “β,” and “γ”), numbers (“1,” “2,” and “3”), and animal icons (dog, cat, and duck) for front‐side illumination and Latin letters (“D,” “E,” and “F”), Greek letters (“δ,” “ε,” and “ζ”), numbers (“4,” “5,” and “6”), and weather icons (sun, moon, and cloud) for back‐side illumination. In total, 24 distinctive intensity or polarization images were engraved on a single device.

Figures 6b,c show the experimentally reconstructed images. The post‐processed results clearly reproduced the intended target intensity and polarization images in good agreement with the numerical predictions (Figure S13). This demonstrates for the first time that it is possible to integrate bidirectionality and OAM holography in a linear optics platform and that the principle can be readily integrated into various optical systems, as it does not rely on nonlinearity.

## 3 Conclusions

We proposed a metasurface‐based platform for total angular momentum‐multiplexed vectorial holography, enabling the generation of independent vectorial holographic images depending on both the spin and orbital angular momenta—equivalently, the TAM state—of the incident light. For a given helical mode, conventional OAM holography has been limited to generating either intensity‐only images for two orthogonal input polarizations or vectorial images for a single input polarization, due to the inherent lack of chirality control in single‐layer metasurfaces. In contrast, our approach based on bi‐layer metasurfaces, which are capable of arbitrary control over anisotropy and chirality, overcomes this limitation and enables the generation of independent vectorial holographic images for each orthogonal TAM input state. The feasibility of the proposed TAM vectorial holography was validated both numerically and experimentally, demonstrating clear advantages over existing single‐layer OAM holography methods. Furthermore, we extended the concept by integrating it with recent advances in vector beam design and bidirectional asymmetric transmission, introducing additional degrees of freedom for input states such as complex coefficients defining vector beams and the direction of illumination.

Looking ahead, the proposed platform can be further improved in three key directions: input states, output functionalities, and metasurface device architectures. From the input perspective, while this work focused on TAM states defined by simple helical modes with integer indices, future implementations could incorporate more complex phase structures beyond conventional OAM modes to represent high‐dimensional structured beams [55, 56, 57, 58, 59, 60, 61, 62]. Multi‐wavelength [63, 64, 65, 66] or multi‐incidence‐angle [33, 34] operations could also enable more diverse forms of multiplexed vectorial holography, further enhancing the information capacity. From the output perspective, although this work focused on far‐field vectorial holography, recent developments in 3D OAM holography [54, 65, 67] suggest that extending our platform to volumetric information could unlock new functionalities in optical data storage, displays, and optical encryption. Finally, from the device perspective, integration with nonlinear metasurfaces could provide intensity‐dependent or frequency‐mixing functionalities, adding new dimensions of light–matter interactions beyond the linear regime [54, 68, 69]. In parallel, active metasurface platforms can introduce dynamic tunability, allowing the real‐time reconfiguration of holographic images in response to external stimuli [70, 71, 72, 73]. In addition, cascading multiple metasurfaces may enable more complex information processing or enhance multiplexing capacity by sequentially manipulating different optical degrees of freedom [74, 75]. Overall, we envision that the TAM vectorial holography platform presented here will serve as a foundation for next‐generation multi‐dimensional optical systems.

## 4 Experimental Section

### 4.1 Fabrication of Bi‐Layer Metasurfaces

The fabrication began with the deposition of a 790 nm‐thick amorphous Si layer on a quartz substrate using plasma‐enhanced chemical vapor deposition (PECVD). For electron‐beam lithography (EBL), a multilayer stack comprising an adhesion promoter (AR 300‐80, Allresist), a resist (AR‐P 6200.04, Allresist), and a conductive polymer (AR‐PC 5090.02, Allresist) was applied by spin coating. Alignment keys for registering the top and bottom layers were patterned through photolithography, followed by electron‐beam (e‐beam) evaporation to deposit 10 nm‐thick Cr and 100 nm‐thick Au, and a subsequent lift‐off process. The nanoposts in the bottom layer were patterned by EBL. A 60 nm‐thick alumina hard mask was patterned using e‐beam evaporation and a lift‐off process. Deep reactive ion etching (RIE) was performed to obtain high‐aspect‐ratio structures with nearly vertical sidewalls. The entire bottom layer was subsequently encapsulated with a 1450 nm‐thick SU‐8 layer via spin coating, serving both as a protective layer and as a planarized base for the top layer. The top 650 nm‐thick amorphous Si layer was then deposited via radio‐frequency sputtering, and the top layer nanoposts were patterned using a fabrication process identical to that of the bottom layer.

### 4.2 Design of Bi‐Layer Metasurfaces

The bi‐layer metasurfaces, which were designed to operate at a wavelength of 915 nm, consisted of a SiO_2_ substrate, Si nanoposts, and an SU‐8 spacer layer (Figure 2b). Each unit cell has a lateral period of 450 nm. The heights of the lower and upper Si nanoposts were 790 and 650 nm, respectively, and the SU‐8 spacer thickness was 1450 nm. To ensure fabrication compatibility, the major and minor axes of the nanoposts were constrained between 100 and 380 nm. Within this dimensional range, the optical response was simulated using finite‐difference time‐domain (FDTD) software provided by Ansys Lumerical Inc. These simulations were used to build a structural library for designing the TAM vectorial holograms. The complex refractive indices used in the simulations were 1.45 (SiO_2_), 3.61 + 0.0066*i* (PECVD‐grown Si), 3.7 + 0.05*i* (sputter‐grown Si), and 1.56 (SU‐8).

### 4.3 Design of TAM Vectorial Holography

The required distributions of parameters (*ϕ*_1M_, *ϕ*_1m_, *θ*_1_, and *θ*_2_) for holograms were optimized using a gradient descent method. For optimization, the loss function for the *i*‐th incidence TAM condition was defined as

(2) $$\begin{array}{cc} L_{i} & =\sum_{p,q}\left\{{\left({\left|E_{pq,x}^{\left(i\right)}\right|}^{2}-{\left|t_{pq,x}^{\left(i\right)}\right|}^{2}\right)}^{2}+{\left({\left|E_{pq,y}^{\left(i\right)}\right|}^{2}-{\left|t_{pq,y}^{\left(i\right)}\right|}^{2}\right)}^{2}\right.\\ & \;+\left.{\left|E_{pq,y}^{\left(i\right)}t_{pq,x}^{\left(i\right)}-E_{pq,x}^{\left(i\right)}t_{pq,y}^{\left(i\right)}\right|}^{2}\right\} \end{array}$$

where the subscripts *p* and *q* represent the indices in the spatial‐frequency (*k_x_* and *k_y_*) domain, $E_{x}^{\left(i\right)}$ and $E_{y}^{\left(i\right)}$ are the *x*‐ and *y*‐components of the output far‐field electric field, respectively, and $t_{x}^{\left(i\right)}$ and $t_{y}^{\left(i\right)}$ represent the corresponding target field components. The first and second terms ensure intensity matching of the *x*‐ and *y*‐polarization components, whereas the third term enforces relative phase matching. The total loss function is given by $L_{\mathrm{tot}}=\sum_{i}L_{i}$. The gradient of *L*_tot_ with respect to the design parameters was obtained using the chain rule, and the parameters were iteratively updated to minimize *L*_tot_. Repeating this process yielded optimal parameter distributions that generated the desired holographic images (refer to Text S6).

### 4.4 Metrics for Evaluating Fidelity in TAM Holography

To evaluate the fidelity of the numerical reconstruction from the optimized design, we employed two complementary metrics: the correlation coefficient, for assessing image similarity, and the cosine similarity of the Stokes parameter distributions, for assessing polarization fidelity.

The correlation coefficient (CC) between the reconstructed image *I* and its corresponding target image *T* was defined as

(3) $$\mathrm{CC}=\frac{\sum_{a,b}\left(I_{a,b}-\bar{I}\right)\left(T_{a,b}-\bar{T}\right)}{\sqrt{\sum_{a,b}{\left(I_{a,b}-\bar{I}\right)}^{2}}\sqrt{\sum_{a,b}{\left(T_{a,b}-\bar{T}\right)}^{2}}}$$

where *I_a,b_* and *T_a,b_* denote the intensities of the (*a, b*)‐th pixel in the reconstructed and target images, respectively, and $\bar{I}$ and $\bar{T}$ are their mean values. This measure was applied across all 12 holographic images (four intensity and eight polarization‐related images).

For polarization fidelity, we computed the cosine similarity (CS) between the reconstructed and target Stokes parameter images, which is defined as

(4) $$\mathrm{CS}=\frac{\vec{S_{r}}\cdot \vec{S_{t}}}{\vec{S_{r}}\vec{S_{t}}}$$

where $\vec{S_{r}}$ and $\vec{S_{t}}$ denotes the reconstructed and target Stokes parameter vectors, which can be derived from the azimuth and elevation images for each output channel.

### 4.5 Optical Characterization

The fabricated metasurfaces were optically characterized using the setup shown in Figure S11. The polarization state of the incident light was controlled using half‐wave and quarter‐wave plates. After transmission through the metasurface, a Fourier plane was formed at the back focal plane (BFL) of the objective lens (MPLFLN 10x, Olympus). A lens (LSB04, Thorlabs) with a focal length of 150 mm was used to project the Fourier plane onto the CMOS camera (CS505MU, Thorlabs). TAM vectorial holograms were analyzed using the Stokes parameters (Text S12), which were derived from four or six intensity measurements under specific polarization configurations, involving a linear polarizer and a quarter‐wave plate placed in front of the CMOS camera [76].

## Author Contributions

J.J. conceived the idea, and J.S. supervised the project. J.J. conducted the theoretical analyses. J.J. and H.K. performed the numerical simulations and designed the samples. J.J. fabricated the samples and characterized them optically. J.J., H.K., and J.S. prepared the manuscript. All authors have accepted responsibility for the entire content of this submitted manuscript and approved its submission.

## Conflicts of Interest

The authors declare no conflicts of interest.

## Supporting information




**Supporting File**: adma72433‐sup‐0001‐SuppMat.docx.

## Data Availability

The data that support the findings of this study are available from the corresponding author upon reasonable request.
