# Supplementary material for: Arbitrary Total Angular Momentum Vectorial Holography Using Bi‐Layer Metasurfaces
Source: Adv Mater. 2026 Feb 8;38(15):e19106. doi: 10.1002/adma.202519106 (PMC12983444; doi:10.1002/adma.202519106)
Supplement: Supplementary file 1 — Supporting File: adma72433‐sup‐0001‐SuppMat.docx. [file ADMA-38-e19106-s001.docx]

**Supporting Information for “Arbitrary Total Angular Momentum Vectorial Holography Using Bi-Layer Metasurfaces”**

*Joonkyo Jung*^1^, *Hyeonhee Kim*^1^ and *Jonghwa Shin*^1,*^

^1^Department of Materials Science and Engineering, KAIST, Daejeon 34141, Republic of Korea.

^*^E-mail: [qubit@kaist.ac.kr](mailto:qubit@kaist.ac.kr)

**Supporting Texts**

**S1. Comparison of achievable functionalities in single-layer and bi-layer metasurfaces**

The structure of a metasurface, together with the underlying phase-encoding mechanisms, determines the range of optical functionalities that can be achieved. Single-layer and bi-layer metasurfaces therefore exhibit distinct capabilities and limitations, even when fabricated from similar materials, as summarized in Figure S1.

The first category consists of single-layer metasurfaces that rely solely on the geometric phase (or Pancharatnam–Berry phase). While this approach provides a robust and efficient means to achieve full 2*π* phase modulation through simple rotation of the constituent structures, it inherently produces conjugate phase responses for right- and left-circularly polarized (RCP and LCP) incident light, as reflected in its Jones matrix. Consequently, only a single scalar hologram can be encoded for either RCP or LCP incidence, and the output polarization is fixed as the conjugate of the input state (*CP/single/scalar holography* in the context of Figure S1).

The second category is based on the combination of geometric phase and propagation phase within a single-layer metasurface, resulting in a Jones matrix that is unitary and symmetric in the linear polarization basis. This approach enables two independent phase modulations for an arbitrary pair of orthogonal polarization states, with the constraint that the output polarization remains the conjugate of the input. As a result, such platforms enable either polarization-multiplexed scalar holography or the generation of a *single* vectorial hologram for a predefined input polarization by coherently combining the two channels (*AP/multiplexed/scalar* or *AP/single/vectorial* holography).

Single-layer metasurface architectures inherently possess mirror symmetry along the depth direction, which imposes symmetry constraints on the accessible Jones matrices and limits the achievable polarization transformations. In contrast, bi-layer architectures intentionally break this mirror symmetry, thereby enabling asymmetric Jones matrices and significantly expanding the available degrees of freedom.

Introducing an additional geometric phase through the second layer—on top of the geometric phase and propagation phase degrees of freedom already present in the first layer—is sufficient to realize an arbitrary unitary Jones matrix. With this extended controllability, a bi-layer metasurface can encode two independent vectorial holograms for an arbitrary pair of orthogonal input polarization states within a single device (*AP/multiplexed/vectorial* holography). Therefore, bi-layer structures constitute a *necessary* condition for realizing polarization–OAM-multiplexed vectorial holography as defined in this work, corresponding to the previously inaccessible block in Figure 1.

**S2. Unitary Jones matrix of bi-layer metasurfaces**

The Jones calculus is a concise and powerful tool for describing the interaction between light and optical systems [1]. In particular, the seminal work [2] demonstrated that highly transmissive single-layer metasurfaces can be approximated as unitary symmetric Jones matrices. Based on this approximation, we suggested in [3] that by employing bi-layer metasurfaces, the symmetry constraint of Jones matrices can be broken, enabling the realization of arbitrary unitary Jones matrices.

A unitary symmetric matrix can be decomposed as $U_{s}=R\left( \theta\right)\left[ \begin{matrix} e^{i\phi_{M}} & 0 \\ 0 & e^{i\phi_{m}} \end{matrix} \right]R\left( -\theta\right)$ where $U_{s}$ denotes a unitary symmetric matrix, $\phi_{M}$ and $\phi_{m}$ are two independent phase delays, and $R\left( \theta\right)$ represents a rotation matrix with angle $\theta$ relative to the *x*-axis. This decomposition indicates that a single-layer metasurface supports two linear eigen-polarization states defined by $\theta$, with pure phase responses of $\phi_{M}$ and $\phi_{m}$ along the respective eigen-polarizations (that is, major and minor axes).

By cascading two layers of such unitary symmetric matrices, the overall Jones matrix *U* of the system can be expressed as

$$\begin{aligned} U=U_{s,2}U_{s,1}=R\left( \theta_{2} \right)\left[ \begin{matrix} e^{i\phi_{2M}} & 0 \\ 0 & e^{i\phi_{2m}} \end{matrix} \right]R\left( -\theta_{2} \right)R\left( \theta_{1} \right)\left[ \begin{matrix} e^{i\phi_{1M}} & 0 \\ 0 & e^{i\phi_{1m}} \end{matrix} \right]R\left( -\theta_{1} \right) \#\left( S1 \right) \end{aligned}$$

where $U$ represents a unitary matrix and the subscripts 1 and 2 represent the bottom and top layers as in Figure 2(b) in the main text, respectively. While a unitary matrix intrinsically has four degrees of freedom, our bi-layer metasurfaces possess six degrees of freedom. Due to these residual degrees of freedom, two parameters can be fixed as constants without loss of controllability over the Jones matrices. For simplicity and to reduce structural complexity, we set $\phi_{2M}=0$ and $\phi_{2m}=\pi$; in other words, the top layer acts as a half-wave plate with spatially varying rotation angles. Under this condition, the Jones matrix can be rearranged as

$$\begin{aligned} U=\left[ \begin{matrix} \cos\left( 2\theta_{2}-\theta_{1} \right) & \sin\left( 2\theta_{2}-\theta_{1} \right) \\ \sin\left( 2\theta_{2}-\theta_{1} \right) & \cos\left( 2\theta_{2}-\theta_{1} \right) \end{matrix} \right]\left[ \begin{matrix} e^{i\phi_{1M}} & 0 \\ 0 & e^{i\phi_{1m}} \end{matrix} \right]\left[ \begin{matrix} \cos\theta_{1} & \sin\theta_{1} \\ -\sin\theta_{1} & \cos\theta_{1} \end{matrix} \right]. \#\left( S2 \right) \end{aligned}$$

**S3. Alignment tolerance of bi-layer metasurfaces**

The alignment tolerance of the bi-layer metasurfaces was evaluated by numerically analyzing the effect of lateral misalignment between the two layers at both the sub-unit-cell and pixel levels. At the sub-unit-cell level, lateral displacements smaller than a single unit cell were introduced under periodic boundary conditions to examine the sensitivity of the locally periodic response (Figure S2). The corresponding Jones matrix responses showed negligible variation over the considered displacement range, indicating that the optical response is largely insensitive to sub-unit-cell misalignment. This behavior is consistent with the weak inter-layer electromagnetic coupling assumed in the Jones matrix-based design model.

In practical devices, the metasurface is non-periodic, with different nanopost configurations appearing at each pixel. In our design, each pixel corresponds to a supercell comprising a 2-by-2 array of unit cells. Pixel level misalignment was therefore evaluated by laterally shifting one layer relative to the other by unit cell-scale displacements as schematically represented in the left column of Figure S3. As the misalignment increases, both the diffraction efficiency and the image quality decrease monotonically, and when the displacement approaches a full-pixel shift, the reconstructed holographic image becomes severely distorted and eventually unrecognizable.

The fabricated samples exhibited lateral alignment errors of approximately 30 nm, as confirmed by the SEM image in Figure 2(c). Such misalignment lies well within the sub-unit-cell regime and therefore has a negligible impact on device performance. These results indicate that the proposed bi-layer metasurface design is tolerant to realistic fabrication misalignment while remaining sensitive to larger, pixel-scale registration errors.

**S4. Dispersive transmission characteristics of bi-layer metasurfaces**

The dispersive transmission characteristics of the bi-layer metasurface were analyzed to assess the spectral robustness of the design around the target wavelength of 915 nm. Although the metasurface is optimized for a single design wavelength, stable performance over a reasonably broad spectral interval is required in practical implementations. To this end, the transmission responses of the nanoposts in both layers were numerically evaluated over a ±20 nm spectral range centered at 915 nm (Figure S4(a)).

For the first layer, all nanopost geometries corresponding to the 36 discrete combinations of phase delays $\phi_{1M}$ and $\phi_{1m}$, each taking six discrete values ($\phi_{1M/m}\in\{0, \frac{\pi}{3}, \frac{2\pi}{3}, \pi, \frac{4\pi}{3}, \frac{5\pi}{3}\}$), were considered. Because exchanging $\phi_{1M}$ and $\phi_{1m}$ produces a geometry rotated by 90°, the transmission responses under *x*-polarized illumination for unrotated nanoposts ($\theta_{1}=0^{\circ}$) fully describe the anisotropic behavior of every structure. As shown in Figure S4(b), the simulated transmission amplitudes exhibit some dispersive degradation due to the intrinsic resonance characteristics of the structures, but both amplitude and phase remain sufficiently uniform within the operating bandwidth.

For the second layer, the dispersive behavior was evaluated in terms of its half-wave plate functionality. While, in the experiments, sputtered amorphous silicon with higher absorption was used due to limitations of the available deposition equipment, the second layer can also be implemented using the same low-loss silicon as the first layer. Therefore, for completeness, both material configurations were analyzed. As shown in Figure S4(c), the polarization-conversion performance of the second layer remains acceptable over the considered spectral range for both cases, despite some degradation due to dispersion.

Within the ±20 nm spectral range around the design wavelength, the phase deviations of both layers are smaller than the discretization error associated with the six-step phase sampling employed in the design. Consequently, the overall holographic performance is expected to remain stable over this spectral range, with only minor degradation.

The transmission efficiency of the bi-layer metasurface can be estimated from the product of the transmissions of the two individual layers. The first layer exhibits an average transmission of approximately 82%, while the second layer shows average transmissions of about 62% for sputtered silicon and 91% for low-loss silicon at the target wavelength of 915 nm. Accordingly, the total transmission of the bi-layer structure is expected to be on the order of 51% and 75% for the two cases, respectively. Although the absolute transmission is lower than unity, the transmission remains sufficiently uniform across different pixels, allowing the bi-layer metasurface to be well approximated by unitary Jones matrices through a global normalization factor in the design process.

**S5. Comparison between OAM multiplexing methodologies**

Conventional OAM holography typically adopts a channel-wise design strategy. For each target image, a phase-only hologram is independently optimized, and OAM selectivity is introduced by encoding the corresponding helical phase factor. The resulting OAM-selective phase profiles associated with different OAM channels are then superposed to form a single complex-amplitude pattern, from which only the phase component is ultimately implemented as an OAM-multiplexed hologram. In polarization-multiplexed implementations, this procedure is performed separately for predefined, spatially homogeneous input–output polarization pairs. Within such schemes, the available optical intensity is pre-assigned among channels—often in equal amounts—and the input and output polarization states function primarily as channel labels. OAM-multiplexed holograms corresponding to different polarization channels are optimized independently, without being actively coupled during the optimization process.

In contrast, the framework adopted in this work performs a joint, gradient-based optimization in which the input polarization–OAM combinations, target holographic images, and desired output vector polarization states are simultaneously incorporated into a single optimization loop. Rather than superposing independently optimized phase profiles, the metasurface is designed by directly optimizing its full Jones matrix response, with loss and gradient contributions aggregated across all TAM channels.

From an optimization perspective, this formulation considers the constraints associated with different channels in a holistic manner during the design process. As a result, inter-channel trade-offs are naturally taken into account within a single optimization problem, allowing the design space to be fully explored without decomposing it into separate, channel-wise subproblems.

**S6. Gradient descent optimization**

Gradient descent optimization was employed to determine the required distributions of design parameters ($\phi_{1M}$, $\phi_{1m}$, $\theta_{1}$, and $\theta_{2}$) for the holograms presented in the main text. If the loss function $L$ is expressed in terms of the far-field electric field component $E^{f}$ as represented in the experimental section of the main text, the gradient can be computed via the chain rule as follows.

First, the derivatives of $L$ with respect to $E^{f}$ can be analytically evaluated, yielding $\frac{\partial L}{\partial E_{pq,x}^{f}}$ and $\frac{\partial L}{\partial E_{pq,y}^{f}}$, where the subscripts $p$ and $q$ represent indices of the spatial frequency domain and the subscripts $x$ and $y$ denote the *x*- and *y*-polarization components of the field.

Then, applying the chain rule, the derivatives of the loss function with respect to the output fields at the metasurface plane is given as

$$\begin{aligned} \frac{\partial L}{\partial E_{ab,\left( x,y \right)}^{n}}=\sum_{p,q} \frac{\partial L}{\partial E_{pq,\left( x,y \right)}^{f}}\frac{\partial E_{pq,\left( x,y \right)}^{f}}{\partial E_{ab,\left( x,y \right)}^{n}} \#\left( S3 \right) \end{aligned}$$

where $E^{n}$ is the output field at the metasurface plane and the subscript *a* and *b* represent the indices of the spatial domain.

Using the discrete Fourier transform, the far-field electric field and the output field at the metasurface plane have the relation of $E_{pq,\left( x,y \right)}^{f}=\mathcal{F}\left( E_{\left( x,y \right)}^{n} \right)_{pq}=\sum_{a,b} E_{ab,\left( x,y \right)}^{n}e^{-i\left( k_{x}^{\left( p \right)}x^{\left( a \right)}+k_{y}^{\left( q \right)}y^{\left( b \right)} \right)}$ where $\mathcal{F}$ represents the discrete Fourier transform, and the superscripts $a$ and $b$($p$ and $q$) also labels indices of the spatial(spatial frequency) domain. From this relation, the derivatives of $E^{f}$ with respect to $E^{n}$ can be written as $\frac{\partial E_{pq,\left( x,y \right)}^{f}}{\partial E_{ab,\left( x,y \right)}^{n}}=e^{-i\left( k_{x}^{\left( p \right)}x^{\left( a \right)}+k_{y}^{\left( q \right)}y^{\left( b \right)} \right)}$. Substituting this expression into Equation S3, the derivatives of $L$ with respect to $E^{n}$ is given as

$$\begin{aligned} \frac{\partial L}{\partial E_{ab,\left( x,y \right)}^{n}}=\sum_{p,q} \frac{\partial L}{\partial E_{pq,\left( x,y \right)}^{f}}e^{-i\left( k_{x}^{\left( p \right)}x^{\left( a \right)}+k_{y}^{\left( q \right)}y^{\left( b \right)} \right)}=N^{2}\left[ \mathcal{F}^{-1}\left\{ \left( \frac{\partial L}{\partial E_{\left( x,y \right)}^{f}} \right)^{*} \right\}_{ab} \right]^{*}\#\left( S4 \right) \end{aligned}$$

where *N* is the number of points in the spatial domain, and the superscript * represents the complex conjugate.

Lastly, the analytical form of derivatives of $L$ with respect to $\phi_{1M}$, $\phi_{1m}$, $\theta_{1}$, and $\theta_{2}$ can be obtained from the Jones matrix described in Equation S2. If the incident light is given as $E_{ab}^{\mathrm{in}}=\left[ \begin{aligned} E_{ab,x}^{\mathrm{in}} \\ E_{ab,y}^{\mathrm{in}} \end{aligned} \right]$, the output field at the metasurface plane is expressed as $\left[ \begin{matrix} E_{ab,x}^{n} \\ E_{ab,y}^{n} \end{matrix} \right]=\left[ \begin{matrix} U_{ab,11} & U_{ab,12} \\ U_{ab,21} & U_{ab,22} \end{matrix} \right]\left[ \begin{aligned} E_{ab,x}^{\mathrm{in}} \\ E_{ab,y}^{\mathrm{in}} \end{aligned} \right]=\left[ \begin{aligned} U_{ab,11}E_{ab,x}^{\mathrm{in}}+U_{ab,12}E_{ab,y}^{\mathrm{in}} \\ U_{ab,21}E_{ab,x}^{\mathrm{in}}+U_{ab,22}E_{ab,y}^{\mathrm{in}} \end{aligned} \right]$. From this expression, the derivatives of $L$ with respect to $\phi_{1M}$, $\phi_{1m}$, $\theta_{1}$, and $\theta_{2}$ can be analytically represented as

$$\frac{\partial L}{\partial\phi_{1M,ab}}=\sum_{x, y} \left( \frac{\partial L}{\partial E_{ab,\left( x,y \right)}^{n}}\frac{\partial E_{ab,\left( x,y \right)}^{n}}{\partial\phi_{1M,ab}}+\frac{\partial L}{\partial E_{ab,\left( x,y \right)}^{n,*}}\frac{\partial E_{ab,\left( x,y \right)}^{n,*}}{\partial\phi_{1M,ab}} \right)$$

$$\frac{\partial L}{\partial\phi_{1m,ab}}=\sum_{x, y} \left( \frac{\partial L}{\partial E_{ab,\left( x,y \right)}^{n}}\frac{\partial E_{ab,\left( x,y \right)}^{n}}{\partial\phi_{1m,ab}}+\frac{\partial L}{\partial E_{ab,\left( x,y \right)}^{n,*}}\frac{\partial E_{ab,\left( x,y \right)}^{n,*}}{\partial\phi_{1m,ab}} \right)$$

$$\frac{\partial L}{\partial\theta_{1,ab}}=\sum_{x, y} \left( \frac{\partial L}{\partial E_{ab,\left( x,y \right)}^{n}}\frac{\partial E_{ab,\left( x,y \right)}^{n}}{\partial\theta_{1,ab}}+\frac{\partial L}{\partial E_{ab,\left( x,y \right)}^{n,*}}\frac{\partial E_{ab,\left( x,y \right)}^{n,*}}{\partial\theta_{1,ab}} \right)$$

$$\begin{aligned} \frac{\partial L}{\partial\theta_{2,ab}}=\sum_{x, y} \left( \frac{\partial L}{\partial E_{ab,\left( x,y \right)}^{n}}\frac{\partial E_{ab,\left( x,y \right)}^{n}}{\partial\theta_{2,ab}}+\frac{\partial L}{\partial E_{ab,\left( x,y \right)}^{n,*}}\frac{\partial E_{ab,\left( x,y \right)}^{n,*}}{\partial\theta_{2,ab}} \right).\#\left( S5 \right) \end{aligned}$$

**S7. Multiplexing capacity and crosstalk**

Although the proposed framework enables the joint optimization of multiple TAM channels within a unified design flow, the achievable multiplexing capacity is subject to several practical constraints. In this section, we first discuss the factors that limit the number of multiplexed channels and then clarify the main origins of crosstalk in the proposed scheme.

*Practical limitations on multiplexing capacity*

Owing to the rich per-pixel degrees of freedom (DoF) and the high spatial resolution inherently provided by metasurfaces, the available design freedom can, in principle, be distributed across multiple channels to encode distinct holographic information. In practice, however, the multiplexing capacity is restricted by several factors.

First, image reconstruction is performed on a discrete two-dimensional sampling grid with a finite pixel spacing in the image plane. Under this constraint, the allowable range of helical mode indices is limited by the sampling interval. Increasing the span of helical mode indices enlarges the ring diameters of non-target doughnut-shaped modes, which leads to stronger overlap between neighboring pixels and enhanced inter-channel crosstalk. To mitigate this effect, one must either restrict the usable range of OAM, thereby reducing the number of multiplexed images, or employ sparse aperture arrays, which in turn lowers the effective image resolution.

Second, although metasurfaces provide rich per-pixel DoF, the total available DoF are finite and must be shared among all multiplexed channels, which introduces an intrinsic trade-off between the number of TAM channels and the achievable fidelity of each channel. The total DoF are determined by the device size and spatial resolution (or unit cell size), which together govern the number of helical modes that can be faithfully supported, the spacing between their indices, and the maximum viable OAM index. As additional channels are introduced, fewer DoF can be allocated to each channel, leading to gradually reduced fidelity and increased crosstalk.

Third, experimental considerations further limit the achievable multiplexing capacity. Fabrication imperfections, such as deviations in nanopost dimensions and rotation angles, and measurement imperfections, including misalignment of polarization optics, become increasingly critical as the number of multiplexed channels increases, thereby imposing a practical upper bound on the channel count.

Within these practical constraints, the proposed framework allows the available design DoF to be carefully distributed across multiple TAM channels, enabling faithful holographic encoding for each channel as long as the overall channel count remains compatible with the metasurface design constraints, as demonstrated in Figure S8(b) for the case of 16 TAM channels.

*Origin of crosstalk*

Residual crosstalk arises from constraints in the metasurface hologram design. For polarization–OAM-multiplexed holography, theoretical vectorial far-field distributions can be defined, from which theoretical Jones matrices are directly derived from the desired input states and target far-field distributions using the Fourier transform-based formulation. These theoretical Jones matrices generally consist of four complex-valued, independent elements.

In practice, to ensure high transmission efficiency, the metasurface is optimized based on the unitary Jones matrices. This discrepancy inevitably introduces errors relative to the theoretical Jones matrices, which constitutes the algorithmic source of crosstalk between TAM channels. Nevertheless, owing to the high design flexibility of the metasurface, the crosstalk can be effectively suppressed during optimization as long as the number of channels remains compatible with the available DoF.

To qualitatively validate the suppression of crosstalk in our devices, we numerically reconstructed intensity images of the optimized design as in Figure S5(a). The target input polarization states are *x*- and *y*-polarizations and target helical mode indices were given as *l* = 1, 2. The top and bottom rows represent raw intensity and filtered intensity images, respectively. Comparing the columns directly reveals the crosstalk between different OAM and polarization channels (e.g., the comparison between the first and second columns shows the crosstalk between polarization channels with the same mode index, and so on). The filtered results explicitly demonstrate that the crosstalk in our designs was sufficiently suppressed and became negligible after removing unwanted OAM modes with doughnut-like profiles.

Experimental imperfections may further degrade device performance. Specifically, unavoidable deviations in structural dimensions and rotation angle of nanoposts during fabrication can modify the local phase and polarization responses, hindering the precise realization of the target Jones matrices. Additionally, misalignment of polarization optics in the optical characterization setup can deteriorate channel separation.

Despite these experimental imperfections, the fabricated samples and measurements confirmed that TAM vectorial holography can still be realized with sufficiently low crosstalk. Figure S5(b) shows the measured intensity images of the VB-multiplexed vectorial holograms, demonstrating experimental robustness. As with the numerical results, comparing the columns reveals crosstalk between polarization and OAM channels. In this case, since VB #1 and VB #2(VB #3 and VB #4) are from the same HOPS, they indirectly reveal the crosstalk between polarization channels. On the contrary, the vector beam states from different HOPSs can show the crosstalk between OAM channels. Note that the blurred images with non-target OAM modes observed in the raw intensity images (top row) are barely seen in the filtered images (bottom row).

**S8. Spatial arrangement of different output channels**

As mentioned in the main text, we separated output channels with different helical mode indices in the far-field domain for clear visualization of the reconstructed holographic images. However, they may also be placed at the same position as reported in [4]. Two output channels corresponding to the input modes with the same helical mode index but orthogonal polarizations were designed to be spatially overlapped. Figure S7 illustrates the spatial configuration of the output channels for TAM, VB-multiplexed, and bidirectional TAM vectorial holograms. Since each output channel has three target images corresponding to the intensity and polarization distributions, therefore, six different target images are located within each spatial-frequency range. For numerical demonstration of TAM vectorial holography with 16 channels, the channels are spatially overlapped based on the sequence as in Figure S7(d).

**S9. Diffraction efficiency**

In OAM-multiplexed holography, the diffraction efficiency is inherently influenced by both the number of encoded helical modes and the aperture used to isolate a target OAM channel. Unlike wavelength- or polarization-multiplexed holography, practical implementations of OAM multiplexing can give rise to multiple coexisting holographic images carried by distinct doughnut-shaped beam profiles, and the desired image is obtained through a filtering process. As a result, the incident optical power is distributed among multiple coexisting doughnut-shaped beams, leading to a reduced efficiency for any single selected channel.

An additional factor affecting the efficiency arises from the aperture array (Figure S9(a)) used to extract the desired holographic image. As illustrated in Figure S9(b), different coexisting doughnut-shaped beam profiles overlap spatially at the image plane, and an appropriately sized aperture is required to transmit only the fundamental region associated with the target channel. When a relatively small aperture (*s*=*s*_1_) is used, contributions from non-target beam profiles are effectively suppressed, enabling high-quality image reconstruction at the cost of reduced diffraction efficiency. Conversely, increasing the aperture size (*s*=*s*_2_) allows a larger fraction of the desired beam to be transmitted, thereby improving efficiency, but it also admits increased contributions from neighboring beam profiles, resulting in stronger crosstalk and degraded image quality.

This efficiency–quality trade-off is exemplified by the numerical results shown in Figure S9(c). For a representative case in which two OAM-multiplexed holographic images are generated at the same location in the spatial frequency domain, a small aperture yields high image quality (CC=0.920, CS=0.915) with a diffraction efficiency of 15.2%. Increasing the aperture size enhances the efficiency to 46.9%, while the image quality is reduced (CC=0.644, CS=0.678) due to increased contributions from non-target doughnut-shaped beam profiles.

These observations indicate that the reduced diffraction efficiency observed in OAM-multiplexed holography primarily originates from (1) the simultaneous generation of multiple holographic images associated with different doughnut-shaped beams and (2) the aperture-based filtering required to suppress crosstalk between them. The resulting efficiency–quality trade-off is therefore intrinsic to OAM-based holographic filtering schemes.

**S10. Post-processing procedure for reconstructing vectorial holographic images**

To reconstruct target holographic images selectively for specific OAM modes, an aperture array was applied to the measured images. Ideally, this aperture array is aligned in the $k_{x}$ and $k_{y}$ axes. However, due to experimental misalignment of the optical measurement setup, the measured results were generally misaligned (rotated and translated) relative to the aperture array. Therefore, before applying the aperture array, the measured images were rotated and translated to properly match the aperture array, and then the aperture array was applied. Figure S10 illustrates the post-processing procedure for one measured image. The filtered images were subsequently used to calculate the Stokes parameters, as described in Text S12.

While we used a preset aperture array to filter out unwanted OAM modes, reconstruction quality can be further improved either through physical calibration or algorithmic compensation of the optical measurement setup. For example, using a reference metasurface with a uniform dot array matched to the aperture array would allow precise registration of aperture positions, thereby enhancing reconstruction accuracy. Alternatively, the aperture array can be algorithmically optimized to account for optical aberrations and distortions inferred from the measured data, enabling compensation for possible optical system-induced imperfections without requiring explicit prior knowledge of these aberrations and distortions.

**S11. Design of input-generating metasurfaces**

For the experimental demonstration of the suggested TAM, VB-multiplexed, and bidirectional TAM vectorial holograms, various input states were required. Specifically, two types of input states were necessary: (1) OAM modes with spatially invariant polarization states and (2) vector beams with spatially varying polarization states. To generate these states, two types of input state-generating metasurfaces were designed. In the experiment, both types of input state-generating metasurfaces were placed immediately before the hologram samples, as shown in Figure S11.

*Polarization-insensitive OAM mode generation*

We used TAM states with various polarization states that remained spatially invariant within each TAM state. To generate these input states, we employed polarization-insensitive OAM mode-generating metasurfaces, which can generate OAM modes without altering the polarization state of the input beam. The required Jones matrix is given as $J=\left[ \begin{matrix} e^{il\varphi} & 0 \\ 0 & e^{il\varphi} \end{matrix} \right]$ where $l$ and $\varphi$ represent the helical mode index and the azimuthal angle in cylindrical coordinates. This can be easily realized using isotropic nanoposts. With the aid of polarization optics, various TAM states can then be prepared.

*Vector beam generation*

As mentioned in the main text, each vector beam state used in this study is given as $\left| \psi\right\rangle=\alpha\left| p_{1},l_{1} \right\rangle+\beta\left| p_{2},l_{2} \right\rangle$. Since we used two orthogonal polarization states ($\left\langle p_{1} | p_{2} \right\rangle=0$) and normalized coefficients ($\left| \alpha\right|^{2}+\left| \beta\right|^{2}=1$) for constructing VB states, the required vector fields can be written as $\left| \psi\right\rangle=\left[ \begin{matrix} \cos\chi e^{i\xi_{x}} \\ \sin\chi e^{i\xi_{y}} \end{matrix} \right]$ with unity magnitude across the design plane, where $\chi$, $\xi_{x}$, and $\xi_{y}$ were uniquely defined by the required polarization and phase distributions. To generate these fields under *x*-polarization illumination, the required Jones matrix is $J=\left[ \begin{matrix} \cos\chi e^{i\xi_{x}} & \sin\chi e^{i\xi_{y}} \\ \sin\chi e^{i\xi_{y}} & -\cos\chi e^{i\left( 2\xi_{y}-\xi_{x} \right)} \end{matrix} \right]$, which is unitary and symmetric. This can be realized using single-layer metasurfaces. Note that a different single-layer metasurface was required for each input vector beam state.

**S12. Optical characterization of the Stokes parameters**

The polarization states of light can be represented using the Stokes parameters. The Stokes parameters are defined as

$$S_{0}=I_{x}+I_{y}=I_{45^{\circ}}+I_{135^{\circ}}=I_{R}+I_{L}$$

$$S_{1}=I_{x}-I_{y}$$

$$S_{2}=I_{45^{\circ}}-I_{135^{\circ}}$$

$$\begin{aligned} S_{3}=I_{R}-I_{L} \#\left( S6 \right) \end{aligned}$$

where $I$ denotes the measured intensity, and the subscripts $x$, $y$, $45^{\circ}$, $135^{\circ}$, $R$, and $L$ represent *x*-polarized, *y*-polarized, 45° linearly polarized, 135° linearly polarized, right-circularly polarized, and left-circularly polarized states. Therefore, these parameters can be quantitatively retrieved by measuring polarized intensity using the optical measurement setup shown in Figure S11. From the Stokes parameters, two spherical coordinates (azimuth and elevation on the Poincaré sphere) can be directly obtained from the following relation:

$$2\psi=atan2\left( \frac{S_{2}}{S_{1}} \right)$$

$$\begin{aligned} 2\chi=\mathrm{atan} \left( \frac{S_{3}}{S_{0}} \right).\#\left( S7 \right) \end{aligned}$$

**
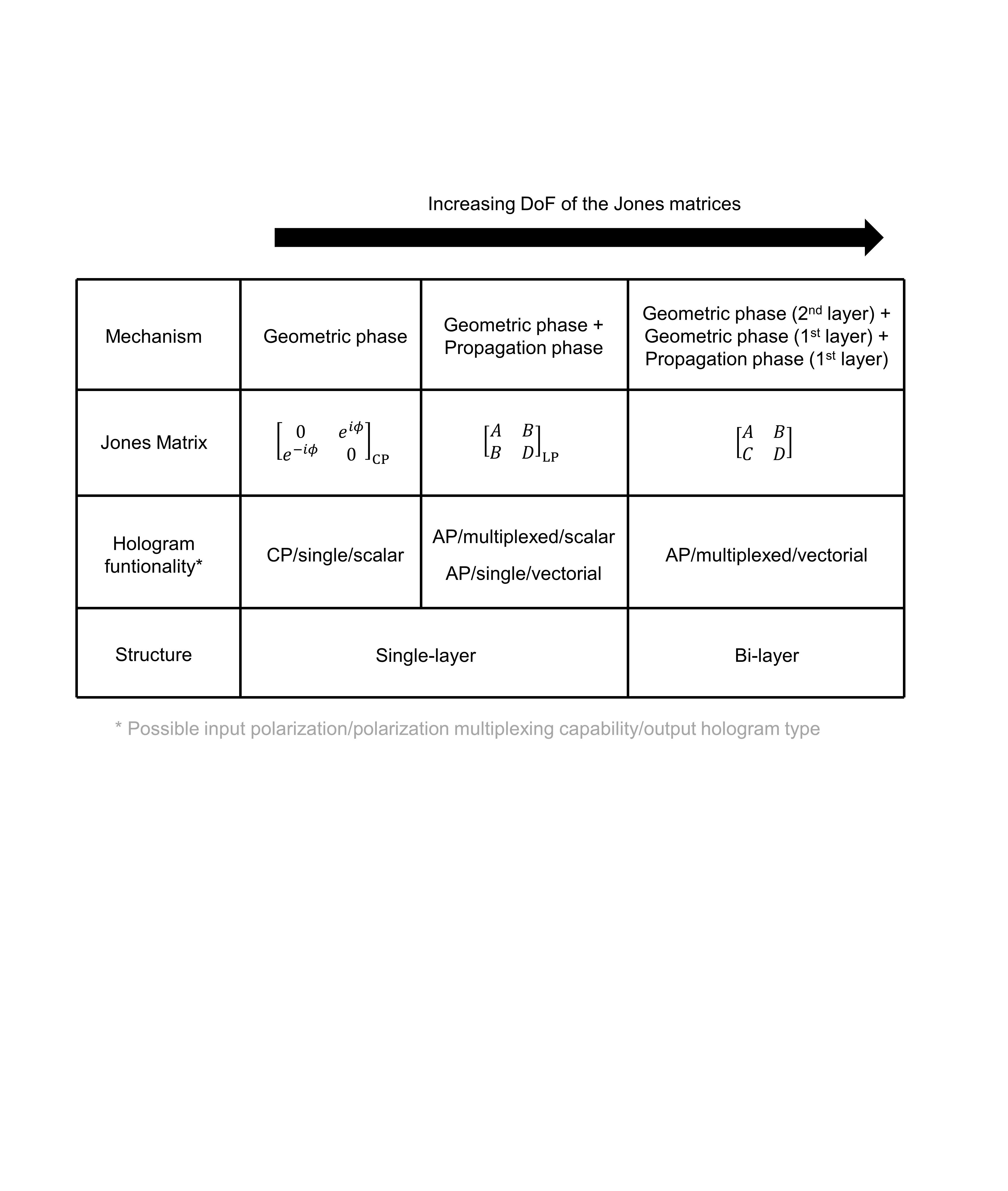
Figure S1. Comparison between single-layer and bi-layer metasurfaces.** CP: circular polarization, AP: arbitrary polarization, LP: linear polarization.

**
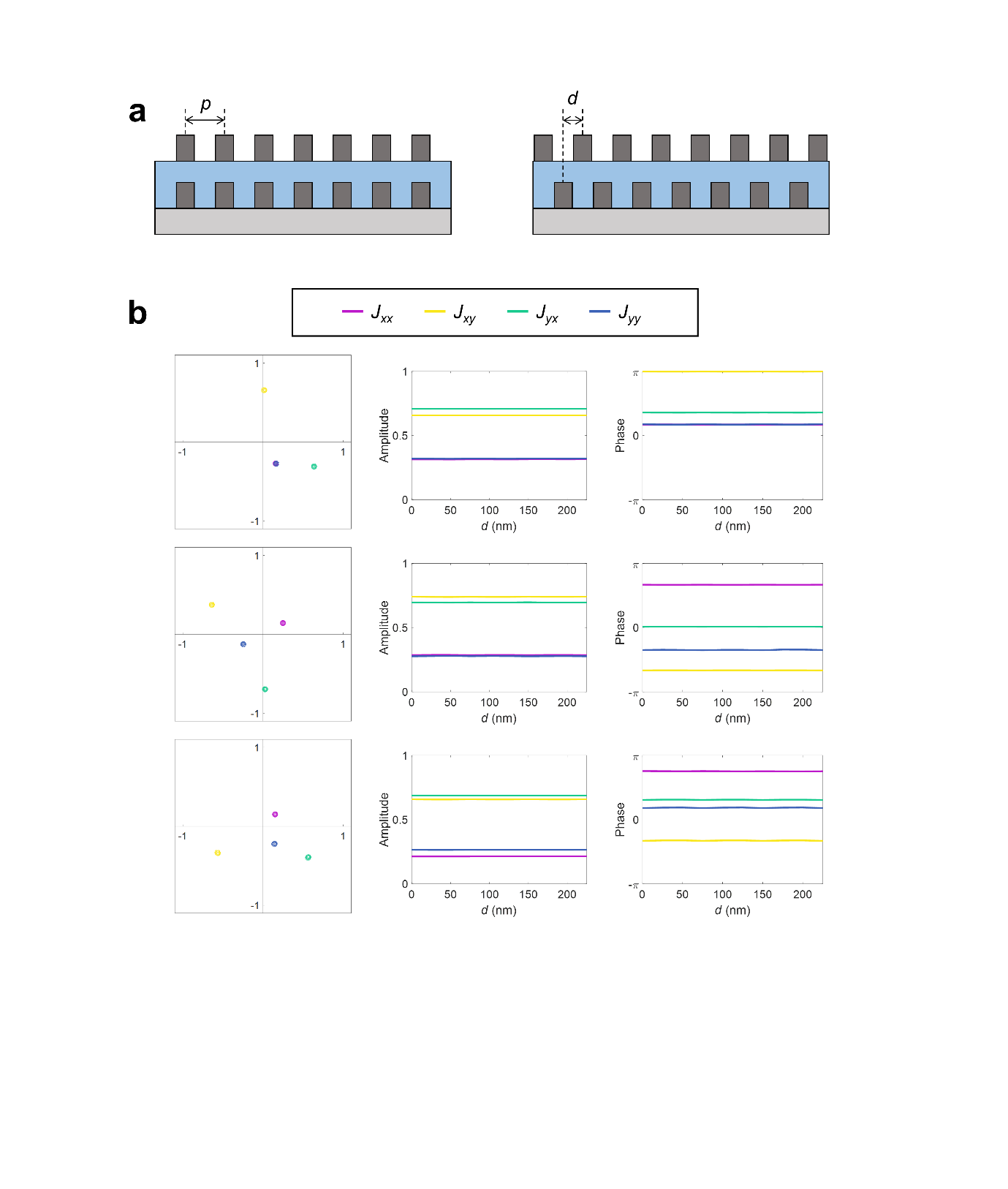
Figure S2. Analysis of misalignment tolerance of bi-layer metasurfaces at the sub-unit-cell level.** (a) Schematics of perfectly aligned (left) and laterally misaligned (right) structures. *p*: period (450 nm). *d*: misalignment (0–225 nm). (b) Analysis of the Jones matrices for three representative structures as a function of misalignment. Each row corresponds to the FDTD simulation results for a specific structure. In the left panel, the elements of the Jones matrices retrieved from the FDTD simulations are plotted on the complex plane as circles with increasing radii, corresponding to increasing misalignment.

**
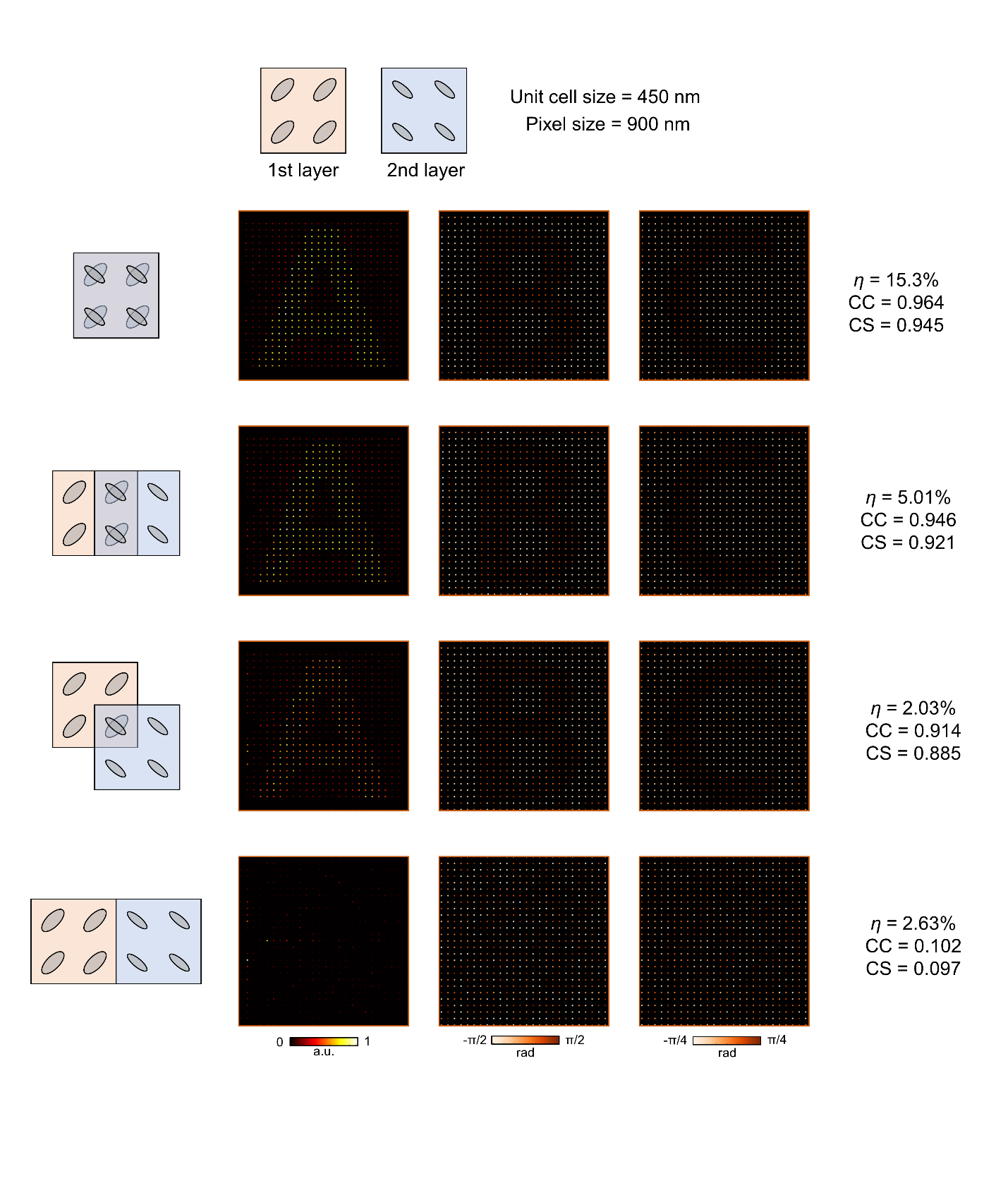
Figure S3. Analysis of misalignment tolerance of bi-layer metasurfaces at the pixel level.** The left column schematically illustrates pixel-level misalignment. In the ideal case, the two layers are perfectly aligned to form a single pixel, as shown in the first row. The right column presents quantitative analyses of the averaged diffraction efficiencies (*η*), correlation coefficients (CC), and cosine similarity (CS) as defined in the main text.

**
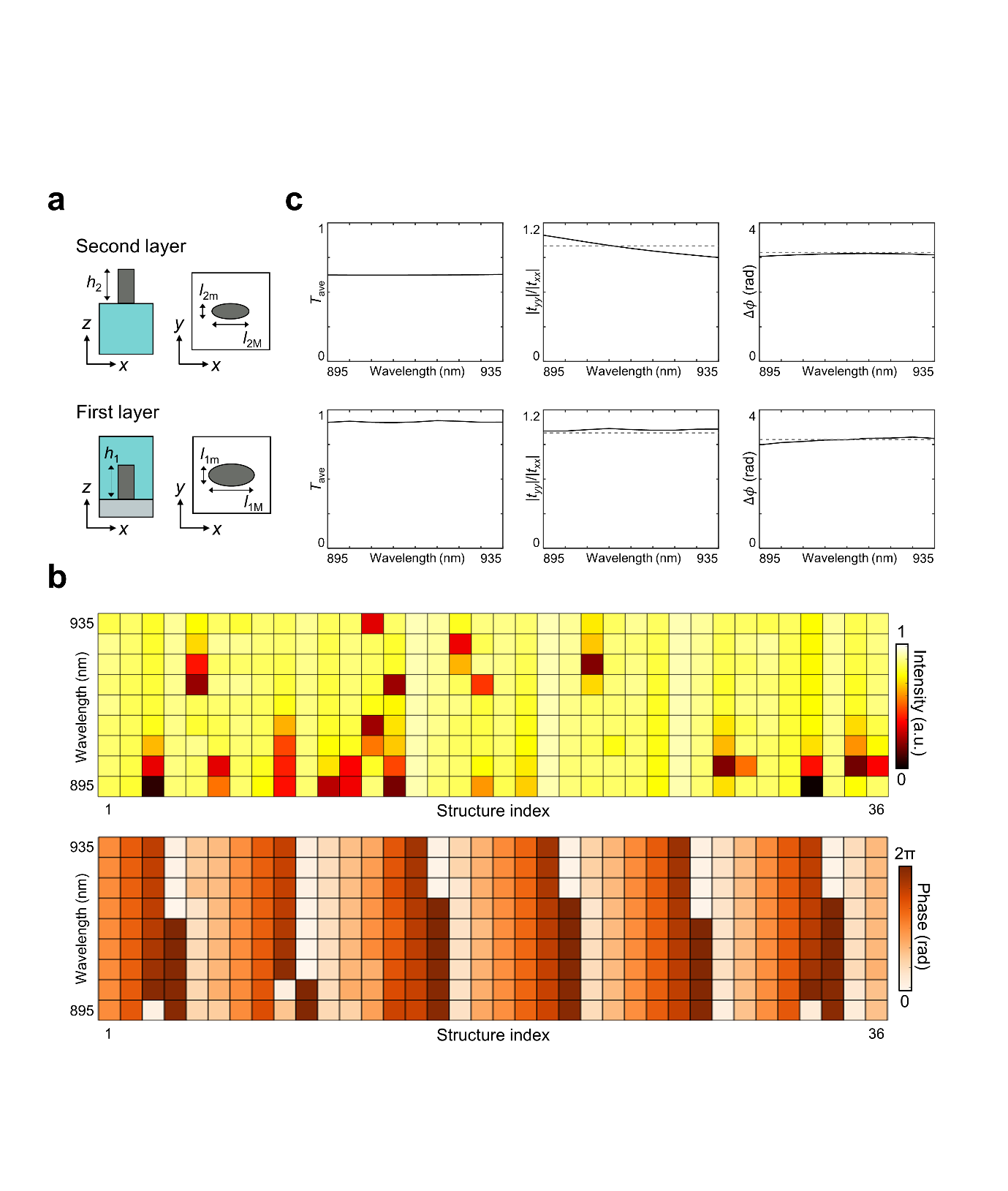
Figure S4. Analysis of the dispersive transmission characteristics of the constituent nanoposts.** (a) Schematics of the nanopost in each layer, aligned along the *x*-axis. (b) Dispersive transmission characteristics of the 36 constituent nanopost geometries used in the first layer. The transmission coefficients are retrieved from the *x*-polarized output under *x*-polarization incidence (*t_xx_*). (c) Dispersive characteristics of the nanoposts in the second layer. The first and second rows correspond to the results of sputtered silicon and low-loss silicon, respectively. For ideal half-wave plate operation, the magnitudes of the transmission coefficients along *x*- and *y*-directions are equal (*t_yy_*/*t*_xx_=1), and the relative phase, defined as $\Delta\phi=\phi_{xx}-\phi_{yy}$ where $\phi_{xx/yy}$ denotes the phase of *t_xx_*_/_*_yy_*, is equal to *π_._* The ideal conditions are indicated by gray dotted lines.

**
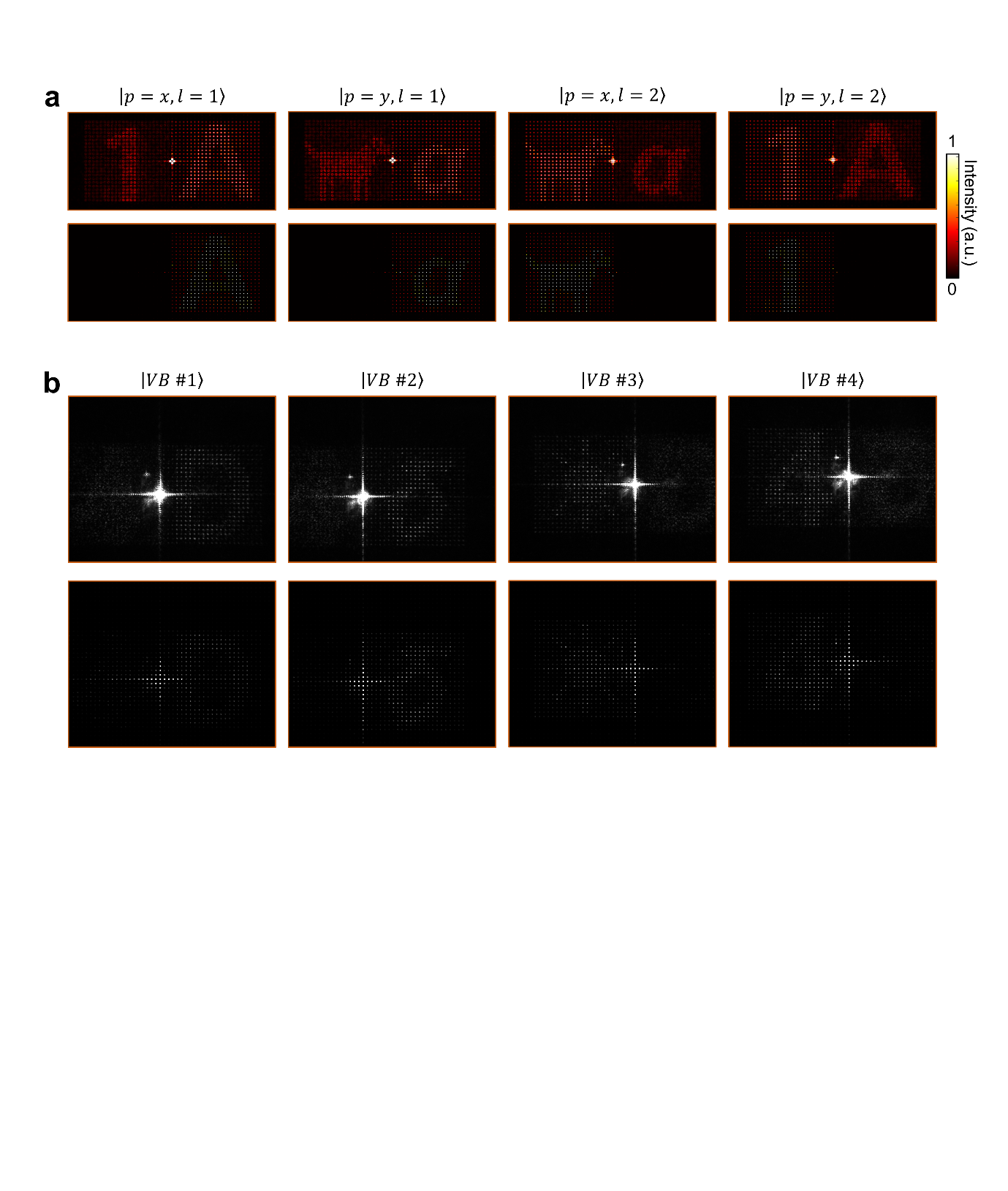
Figure S5. Crosstalk analysis.** (a) Numerical verification of polarization–OAM channel crosstalk in TAM vectorial holography. Top: raw intensity images; bottom: filtered intensity images for each input TAM state. (b) Experimental verification polarization–OAM channel crosstalk in VB-multiplexed vectorial holography. Top: raw intensity images; bottom: filtered intensity images for each input VB state.

**
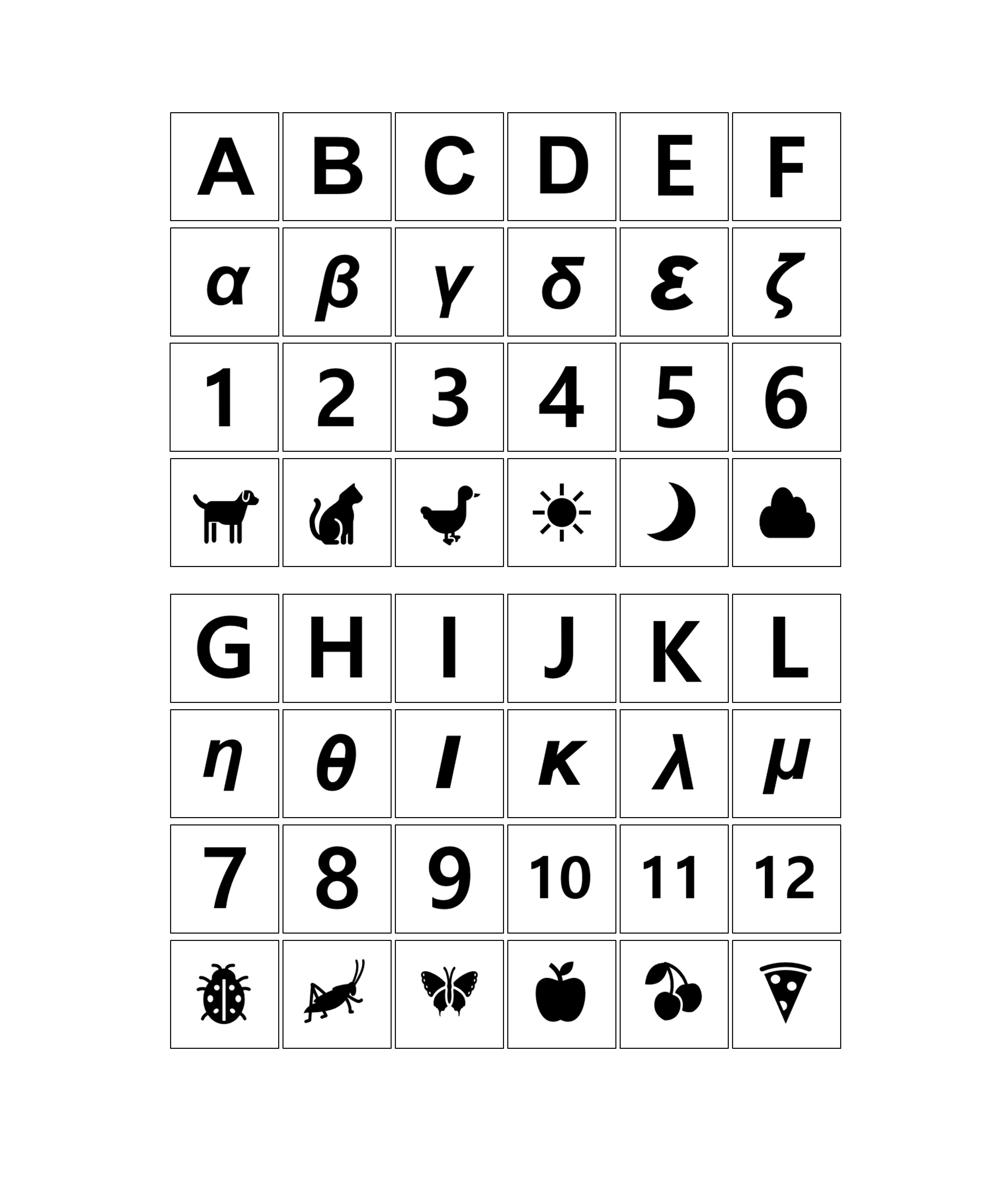
Figure S6. Ground truth images.**

**
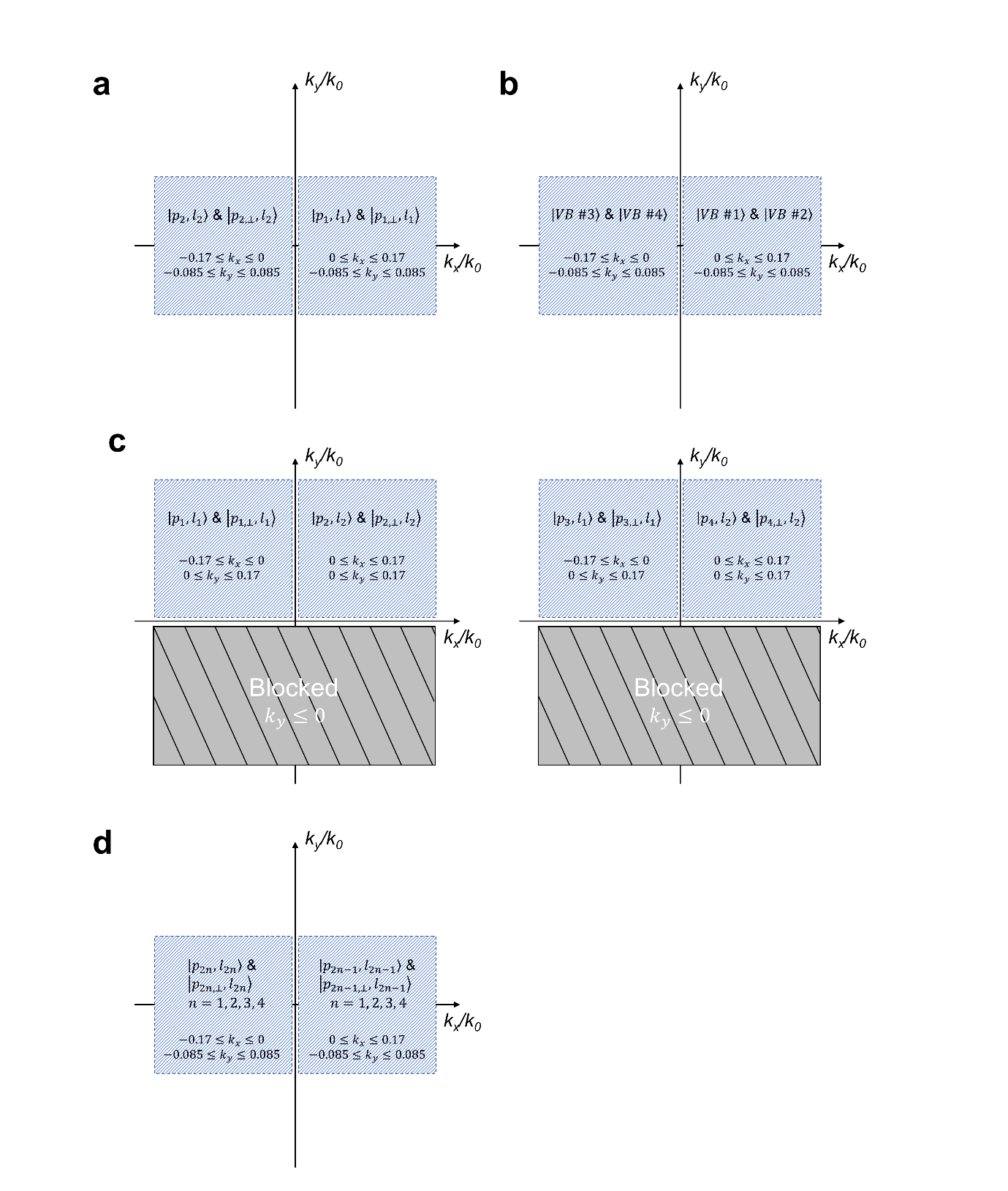
Figure S7. Spatial separation of output channels in the spatial frequency domain.** (a) Configuration for TAM vectorial holography. (b) Configuration for VB-multiplexed vectorial holography. (c) Configuration for bidirectional TAM vectorial holography. The right and left panels correspond to the front- and back-side illumination conditions. (d) Configuration for TAM vectorial holography with 4/8/12/16 TAM channels in Figure S8.

**
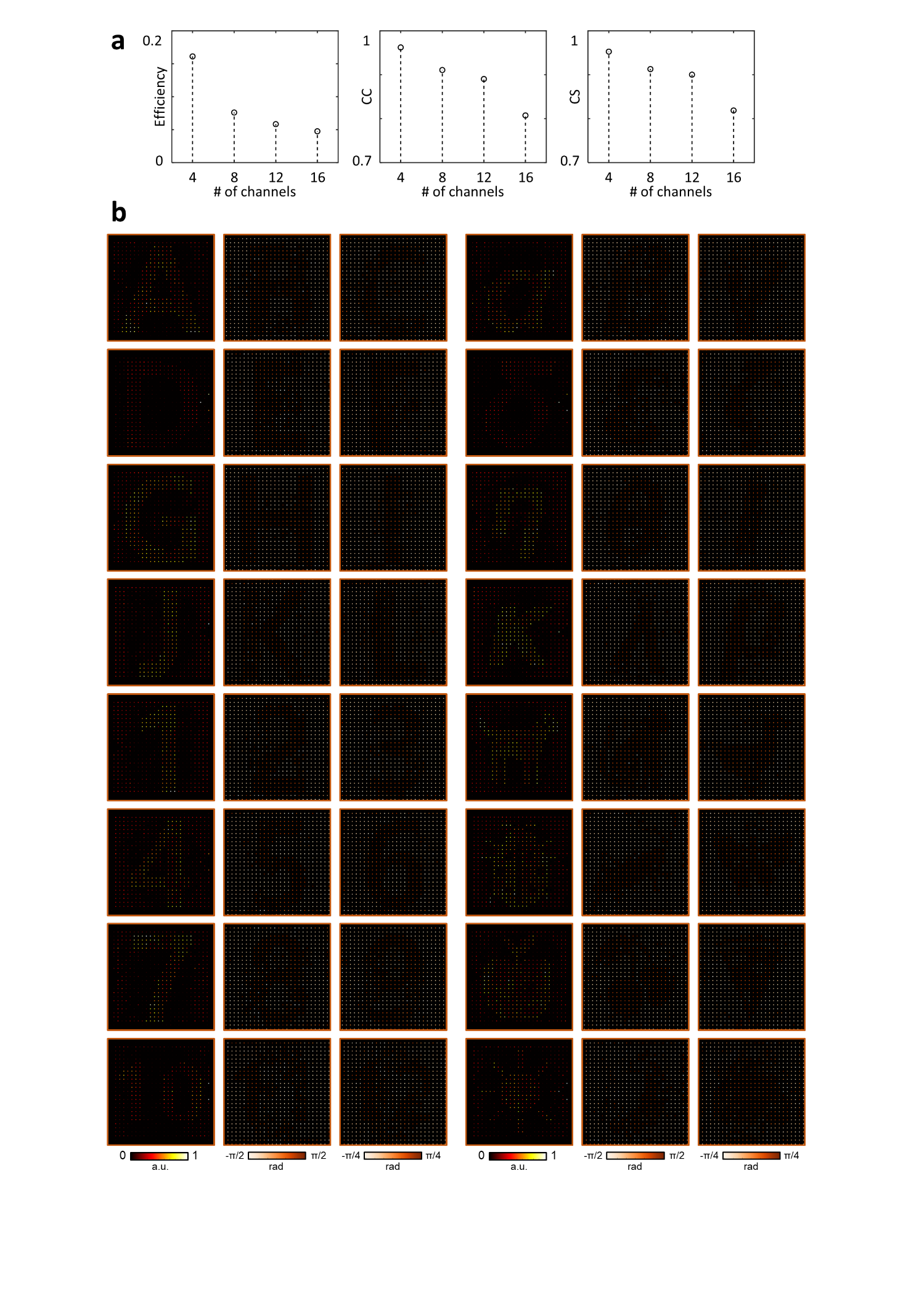
Figure S8. Scalability of TAM vectorial holography.** (a) Quantitative analyses. CC: correlation coefficients. CS: cosine similarity. (b) Numerical demonstrations.

**
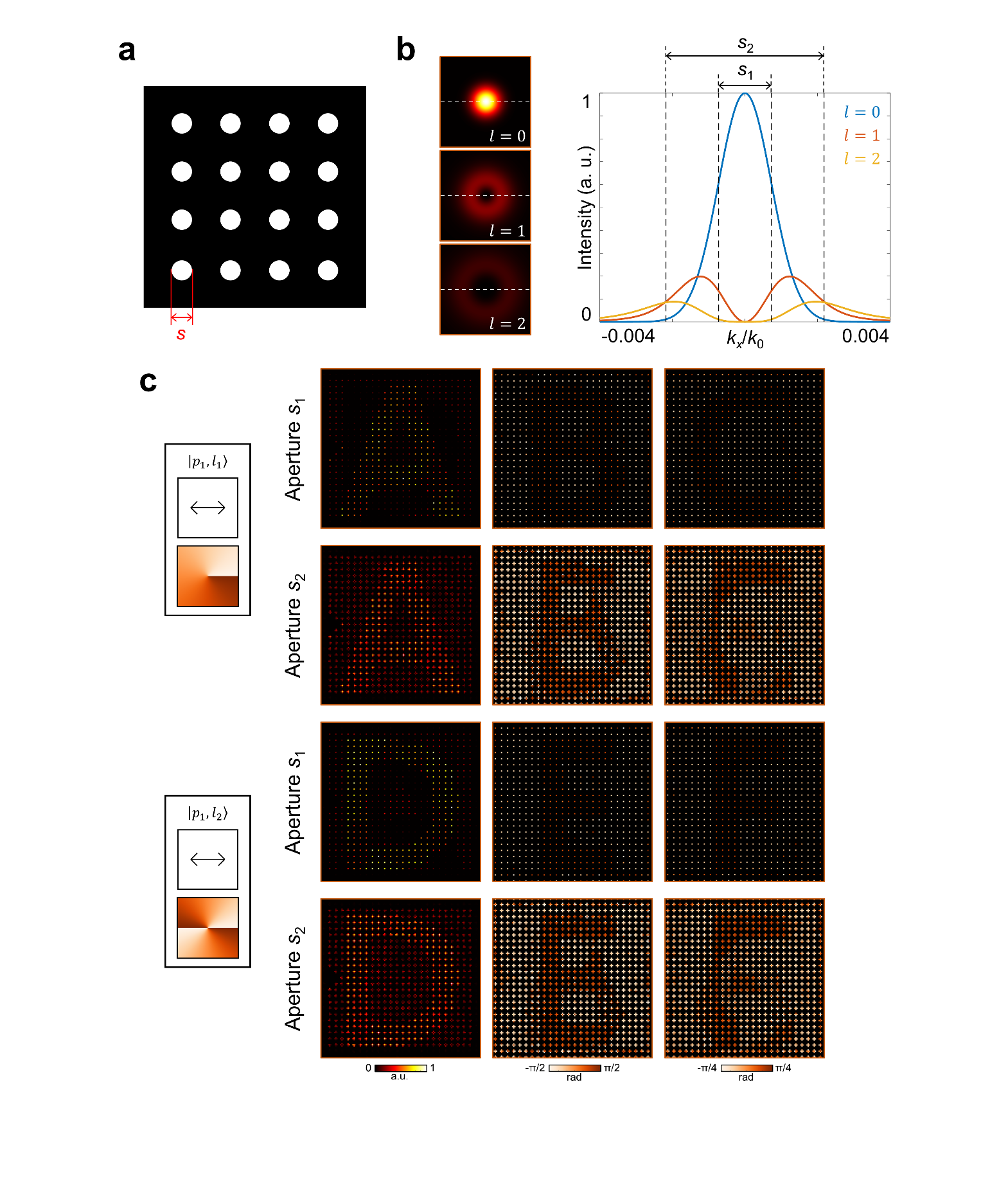
Figure S9. Analysis of device performance as a function of aperture size.** (a) Schematics of the aperture array. (b) OAM beam profiles corresponding to different helical mode indices. The right panel shows the intensity profiles extracted along the white line indicated in the left panels. (c) Numerical evaluation of the effect of aperture size on holographic performance.

**
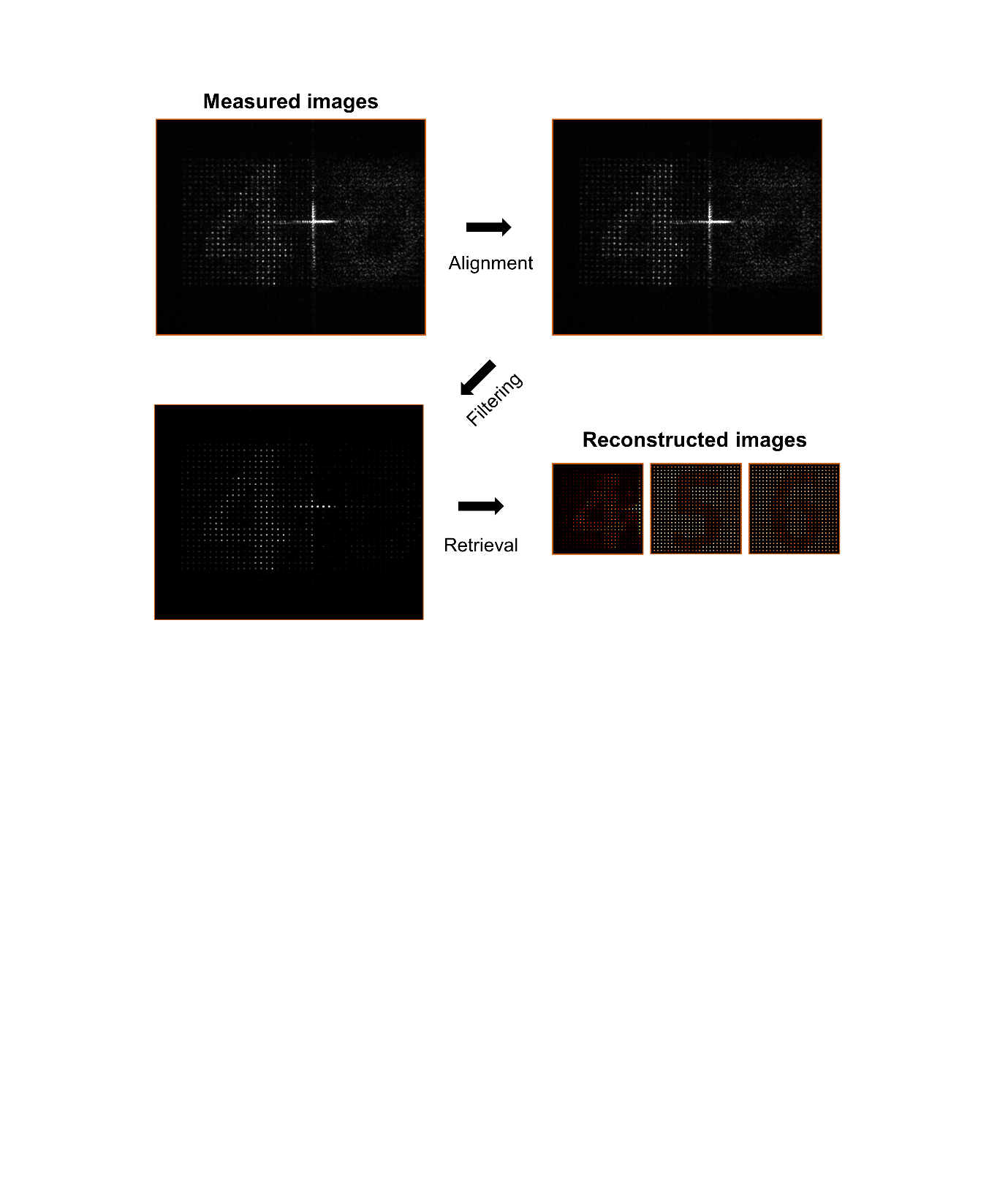
Figure S10. Post-processing procedure.** Measured results were aligned (rotated and translated), filtered by the aperture array, and then used to retrieve the Stokes parameters.

**
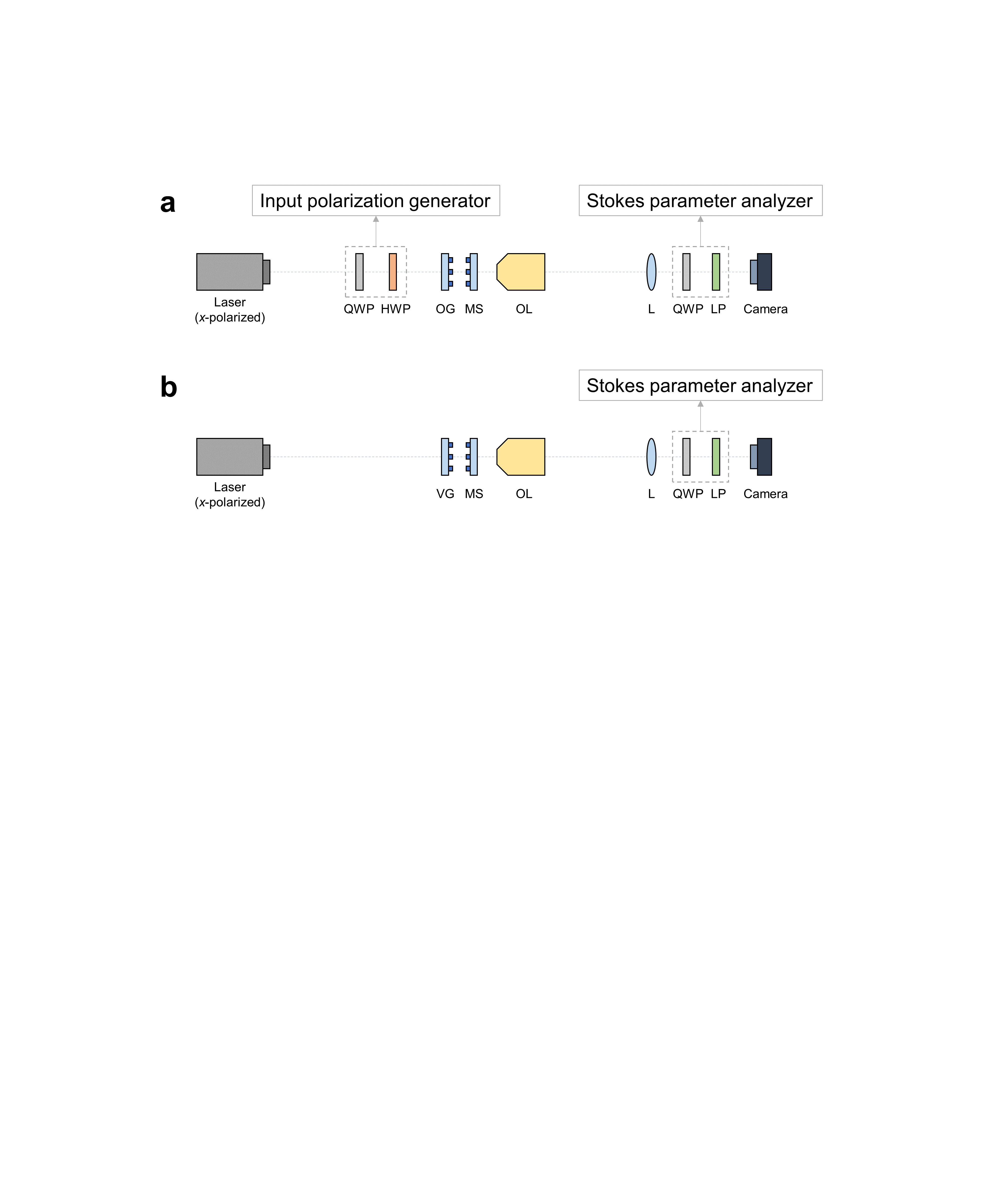
Figure S11. Optical characterization setup.** Experimental setup for measuring far-field holographic images with (a) TAM states and (b) VBs as input. The holographic images are formed at the back focal plane of the objective lens and relayed to the camera using an additional lens placed to transfer the images to the camera. QWP: quarter-wave plate; HWP: half-wave plate; MS: metasurfaces; OL: objective lens; L: lens; LP: linear polarizer; OG: OAM input-generating metasurfaces; VG: VB input-generating metasurface.

**
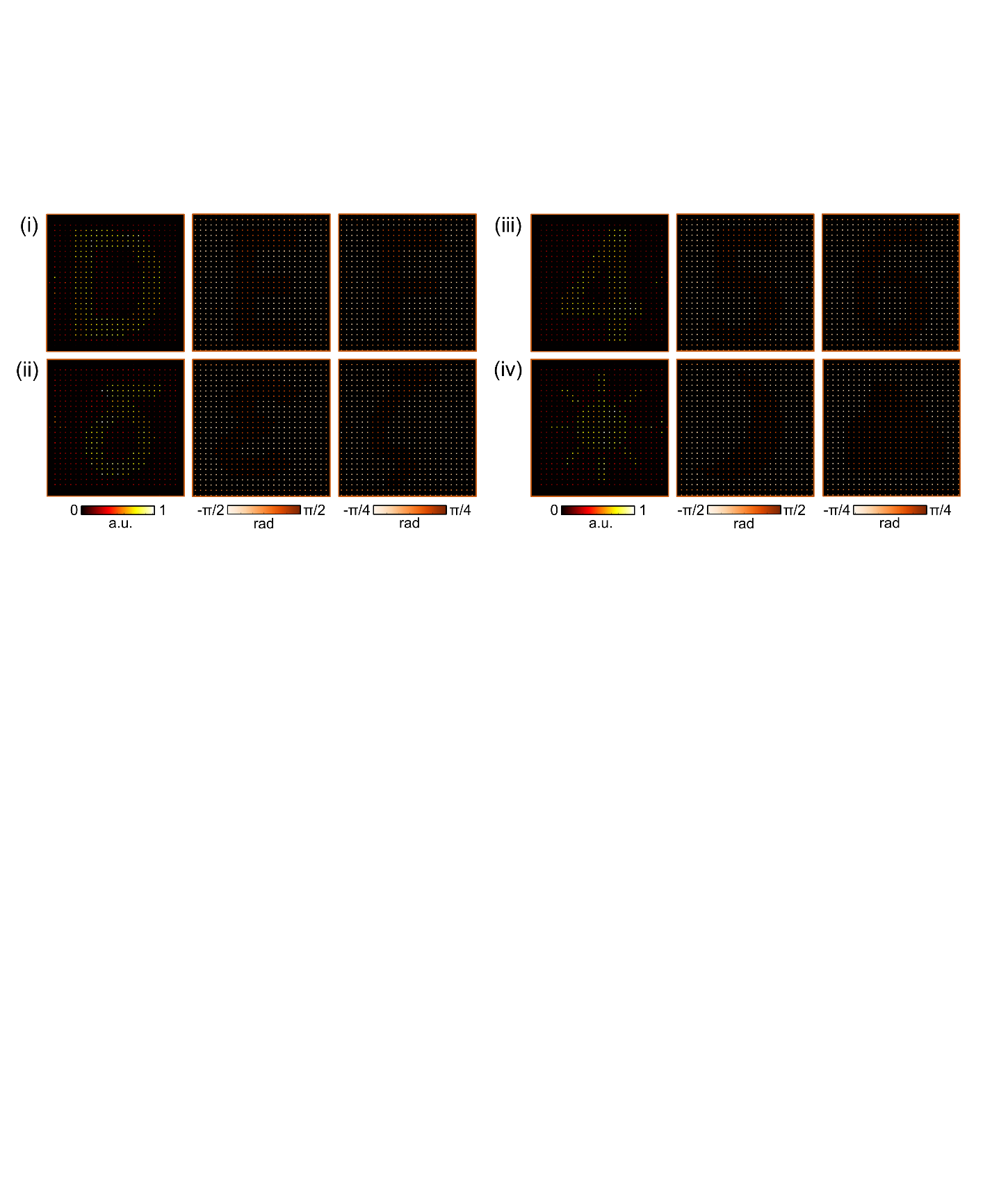
Figure S12. Numerical demonstration of VB-multiplexed vectorial holography.**

**
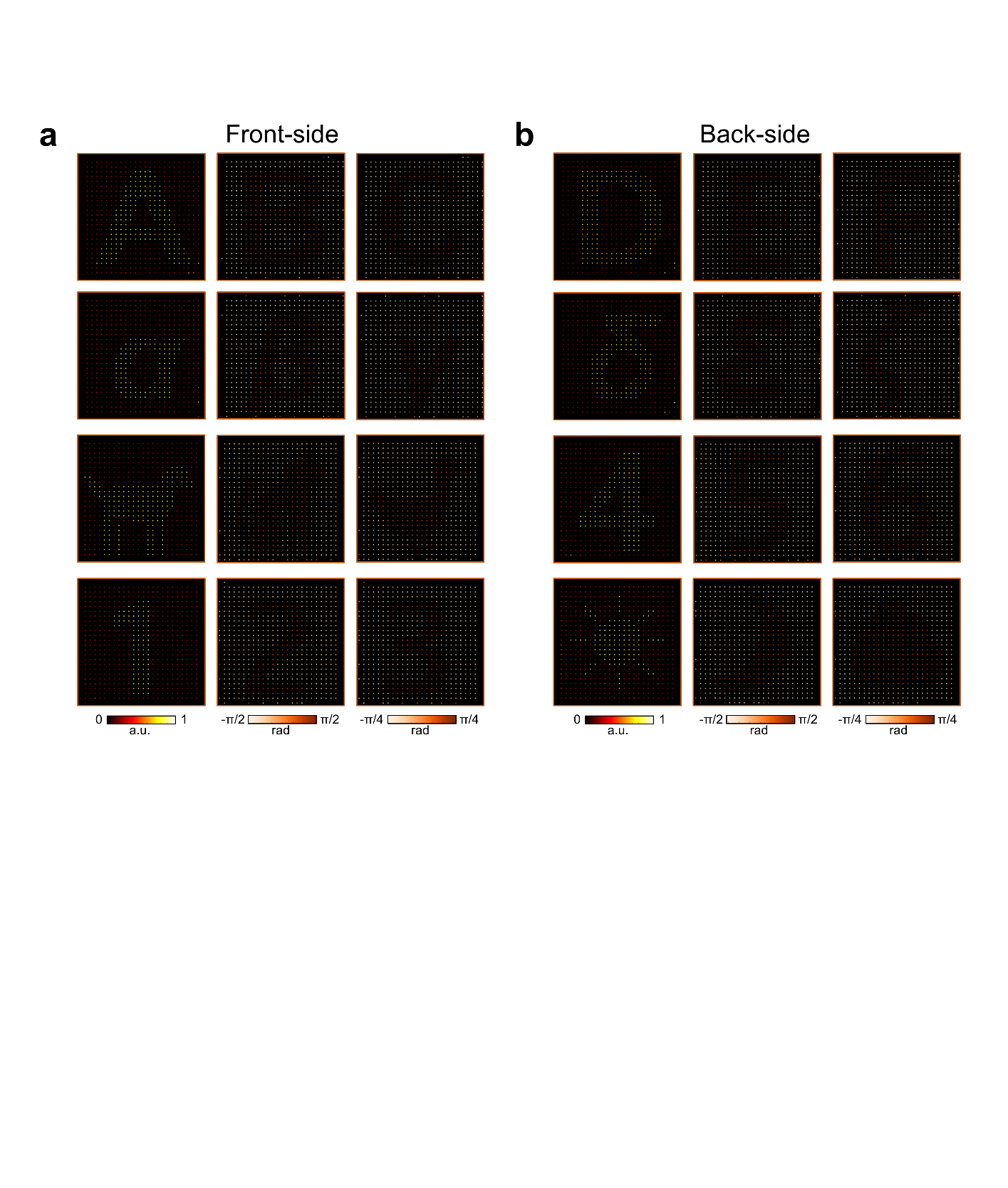
Figure S13. Numerical demonstration of bidirectional TAM vectorial holography.** (a) Reconstructed images for the front-side illumination. (b) Reconstructed images for the back-side illumination.

**References**

1. C. Menzel, C. Rockstuhl, and F. Lederer, *Advanced Jones calculus for the classification of periodic metamaterials.* Physical Review A—Atomic, Molecular, and Optical Physics, 2010, **82**, 053811.

2. A. Arbabi, Y. Horie, M. Bagheri, and A. Faraon, *Dielectric metasurfaces for complete control of phase and polarization with subwavelength spatial resolution and high transmission.* Nature Nanotechnology, 2015, **10**, 937.

3. T. Chang, J. Jung, S.H. Nam, H. Kim, J.U. Kim, N. Kim, S. Jeon, M. Heo, and J. Shin, *Universal metasurfaces for complete linear control of coherent light transmission.* Advanced Materials, 2022, **34**, 2204085.

4. X. Fang, H. Ren, and M. Gu, *Orbital angular momentum holography for high-security encryption.* Nature Photonics, 2020, **14**, 102.
